# Uncertainty-aware fourier-encoded attentive Bi-GRU for non-invasive blood glucose estimation from multimodal PPG and GSR signals

**DOI:** 10.3389/fmedt.2026.1979468

**Published:** 2026-10-28

**Authors:** Allam Jaya Prakash, Herbert F. Jelinek, Mohamed Atef

**Affiliations:** 1 Department of Electrical and Communication Engineering, United Arab Emirates University, Al Ain, United Arab Emirates; 2 Department of Electronics and Communication Engineering, Birla Institute of Technology Mesra, Ranchi, India; 3 Department of Biomedical Engineering and Biotechnology, Khalifa University, Abu Dhabi, United Arab Emirates; 4 Zayed Center for Health Sciences, United Arab Emirates University, Al Ain, United Arab Emirates

**Keywords:** blood glucose estimation, galvanic skin response, multimodal sensor fusion, non-invasive monitoring, photoplethysmography

## Abstract

Noninvasive estimation of glucose from wearable physiological signals remains challenging because sensor–glucose relationships vary across individuals and point estimates do not convey reliability. This article presents an uncertainty-aware regression framework that combines photoplethysmography and galvanic skin response. Synchronized signal segments are transformed using deterministic harmonic Fourier features and processed by a dense projection, a bidirectional gated recurrent unit with additive attention, and a multilayer regressor. Training uses a composite objective comprising mean absolute error, input-gradient smoothness, and a soft constraint on standardized outputs, while Monte Carlo dropout estimates model uncertainty during inference. The framework was evaluated on PhysioCGM recordings from 10 adults with type 1 diabetes, with continuous glucose monitor measurements used as reference values. A random sample-wise 80/20 split yielded a mean absolute error of 3.09 mg/dL, a root mean-square error of 4.72 mg/dL, and a coefficient of determination of 0.960; 96.3% and 99.1% of predictions were located in Clarke Error Grid Zone A and Zones A+B, respectively. In contrast, ten-fold leave-one-subject-out evaluation yielded a mean absolute error of 16.75 mg/dL with a between-fold standard deviation of 0.44 mg/dL and substantially weaker agreement in the hypoglycemic range. These findings indicate strong within-cohort interpolation but limited generalization to unseen individuals and underrepresented glycemic extremes. Larger participant-disjoint studies and calibrated uncertainty estimates are required before clinical or wearable deployment.

## 1 Introduction and literature survey

Diabetes is a major global public-health burden. There are 588.7 million adults aged 20–79 years living with diabetes in 2024 and projected to be 852.5 million by 2050 (1). The disease is characterized by sustained dysregulation of blood glucose level (BGL), and its long-term complications—cardiovascular disease, nephropathy, retinopathy, neuropathy, and acute hypo-/hyper-glycemic events—are closely tied to how tightly glucose is monitored and controlled (2, 3). Effective management, therefore, depends on frequent, ideally continuous, knowledge of the patient’s glycemic state so that diet, physical activity, and medication can be adjusted in time. In current clinical and home practice, BGL is obtained either by capillary finger-prick glucometers or by minimally invasive continuous glucose monitors (CGMs) that insert a subcutaneous electrochemical filament. Both approaches share well-documented drawbacks: they are painful and inconvenient, they raise the risk of infection and skin irritation, finger-prick sampling is inherently intermittent and misses rapid excursions, and CGM consumables are costly and frequently inaccessible in low- and middle-income settings (4, 5). These barriers reduce patient adherence and leave dangerous glycemic swings undetected. Consequently, there is strong motivation to develop *non-invasive* and *automatic* BGL estimation methods that are painless, repeatable, and amenable to everyday wearable use.

Among non-invasive modalities, photoplethysmography (PPG) has emerged as one of the most promising, because the same low-cost optical front-end already embedded in smartwatches, rings, earbuds, and smartphones can capture the pulsatile micro-vascular volume changes that are modulated by glucose-dependent variations in blood composition and vascular tone (6, 7). A parallel line of work exploits galvanic skin response (GSR), also called electrodermal activity, which reflects sweat-gland activity governed by the sympathetic nervous system; clinical studies report a measurable association between skin conductance and glycemic and autonomic state in diabetic subjects (8, 9). However, each single modality is individually fragile: PPG is corrupted by motion artefacts, perfusion changes, and skin-tone dependence, whereas GSR is confounded by stress, ambient temperature, and hydration. This complementary fragility motivates *multimodal fusion*, in which PPG and GSR (optionally with auxiliary vitals) are combined so that the strengths of one modality compensate for the weaknesses of the other, improving robustness and accuracy (10–12).

### 1.1 Non-invasive estimation using PPG alone

PPG-based glucose estimation is the most extensively studied non-invasive route, as comprehensively surveyed in (6, 7). Zhang et al. (13) extracted Gaussian-fitted morphological features from smartphone-camera PPG and classified glycemic level with machine learning, establishing the feasibility of commodity-sensor sensing. Islam et al. (14) regressed BGL from smartphone PPG using classical learners, while Gupta et al. (15) proposed an all-purpose PPG pipeline emphasizing robust pre-processing and feature design. Deep models have since improved accuracy: Zeynali et al. (16) reported a deep network evaluated across diverse patient profiles, and Hammour and Mandic (17) demonstrated an in-ear PPG monitor achieving high clinical-zone agreement on the Clarke error grid. Imaging- and camera-based variants extend PPG to contactless acquisition (18), and ensemble frameworks have been benchmarked for BGL prediction (19). Despite this progress, purely PPG-driven estimators remain sensitive to motion, perfusion, and inter-subject variability, which limits standalone reliability.

### 1.2 Estimation using GSR and other single signals

A complementary body of work exploits electrodermal and other single-channel physiological signals. Prajapati et al. (8) established GSR as a marker of autonomic dysfunction in type 2 diabetes, and bioelectrical skin-impedance studies relate conductance to physiological state (9). Contactless GSR sensing using mmWave radar has also been demonstrated (20), broadening acquisition options. GSR/biofeedback interventions in diabetic patients further confirm a physiological coupling between electrodermal activity and glycemic–autonomic regulation (21). Other single-signal routes include ECG-based estimation (22) and electromagnetic/RF biosensing (23), while broad reviews catalogue emerging non-invasive sensor generations (3, 5). Because electrodermal signals are strongly modulated by stress, temperature, and hydration, GSR-only estimation is typically used for coarse hypo-/hyper-glycemia detection rather than fine-grained regression (4).

### 1.3 Multimodal fusion for improved accuracy

The limitations of any single channel have driven the development of multimodal fusion. Mahmud et al. (10) built a portable multi-sensor wearable for glucose estimation, and Prabha et al. (24) combined wristband PPG with physiological parameters to improve accuracy. Recent systems fuse PPG, ECG, EDA/GSR, skin temperature, and motion with cloud dashboards (11, 25), and the open PhysioCGM dataset provides synchronized ECG/PPG/EDA recordings with CGM ground truth specifically to enable multimodal non-invasive estimation (12). The stress–glucose interaction has been modeled with recurrent networks that jointly exploit emotional and physiological cues (26, 27), and PPG/GSR are routinely combined in adjacent affective-computing tasks such as stress and fatigue detection (28–31). On the modeling side, attention-augmented bidirectional recurrent integrators have achieved state-of-the-art continuous glucose monitoring (32), GRUs provide an efficient recurrent backbone for sequence modeling (33), Fourier feature mappings let networks fit high-frequency low-dimensional functions (34), and MC-dropout yields practical Bayesian uncertainty (35). The proposed method draws these threads together: it fuses PPG and GSR features, encodes them with Fourier features, mixes them with an attentive Bi-GRU, regularizes the mapping using smoothness and standardized-output constraints, and quantifies uncertainty—directly addressing the accuracy and trust gaps left by prior single-modality and point-estimate approaches. Beyond the choice of modality, two further requirements are essential for clinical trust. First, the regressor must capture the nonlinear, high-frequency relationship between morphological signal features and glucose without overfitting; recent evidence shows that explicit frequency-aware encodings and attentive recurrent models substantially improve low-dimensional physiological regression (32, 34). Second, a clinical decision-support tool should report not only a point estimate but also an uncertainty-aware measure of *predictive uncertainty*, so that unreliable readings can be flagged rather than acted upon blindly (35). Most prior non-invasive glucose estimators address at most one of these requirements. The method proposed here unifies multimodal PPG–GSR fusion, Fourier feature encoding, attentive bidirectional recurrent mixing, composite regularization, and Monte-Carlo (MC) dropout uncertainty within a single architecture. The principal contributions of this work are as follows.

- We propose PI-Uncertainty Fourier-BiGRU-NN, an uncertainty-aware regression network for non-invasive BGL estimation that fuses PPG- and GSR-derived features and couples a deterministic harmonic Fourier positional encoding with a dense projection, a bidirectional GRU, and additive attention pooling.
- We design a composite training objective combining a mean-absolute-error data term, an input-gradient smoothness prior that enforces a smooth sensor-to-glucose map without assuming any temporal ordering, and a soft standardized-output range constraint on the predicted z-score. We integrate MC-dropout scale estimation followed by split-conformal calibration to obtain per-sample predictive standard deviations and empirical 95% prediction intervals, enabling uncertainty-aware glucose estimation rather than relying only on point predictions.
- We provide a comprehensive evaluation—scatter/agreement, Bland–Altman, error-vs.-actual, absolute-error distribution, uncertainty–error analysis, Clarke Error Grid, permutation importance, SHAP attribution, and ablation experiments—demonstrating an MAE of 3.09±0.9110 mg/dL, RMSE of 4.72 mg/dL, and $R^{2}=0.960$ on a stratified held-out split, complemented by a Ten-fold leave-one-subject-out (LOSO) MAE 16.75 mg/dL that explicitly characterizes the harder, more clinically relevant inter-subject generalization gap.

The remainder of this paper is organized as follows. Section 2 details the proposed methodology with the mathematical formulation of every stage, the architecture, the regularized loss, and the training/inference algorithm. Section 3 presents the experimental setup and results. Section 4 presents ablation analysis, discussion, and limitations, and Section 5 concludes.

## 2 Proposed methodology

The proposed pipeline maps a vector of measured PPG- and GSR-derived features to a glucose estimate together with an MC-dropout-based predictive uncertainty. The overall processing flow is summarized in Figure 1. Measured features are min–max normalized, Fourier-encoded, and standardized before being passed to the network.

**Figure 1:**
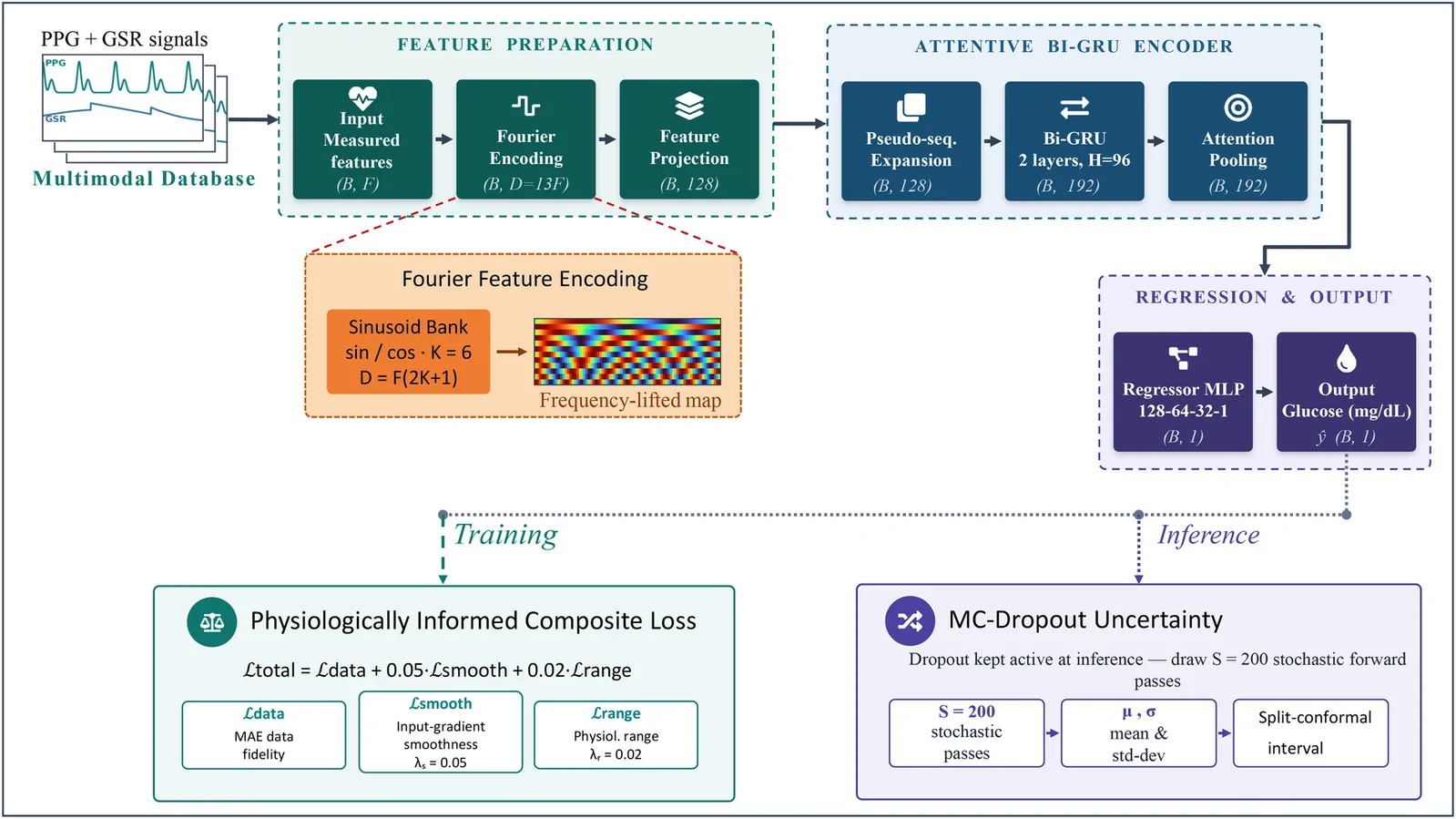
Proposed physiologically informed uncertainty-aware Fourier Bi-GRU architecture for glucose prediction using fused PPG and GSR features.

### 2.1 Feature acquisition and normalization

The detailed architecture of the deep learning model, together with its other principal stages, is shown in Figure 2. Let $x_{\mathrm{raw}}\in R^{D}$ denote the vector of D measured features derived from the synchronized PPG and GSR channels. The morphological descriptors include peak amplitude, pulse interval, rise time, fall time, pulse width, waveform area, local GSR changes, response magnitude, and recovery-related measures; the statistical descriptors include mean, variance, standard deviation, skewness, kurtosis, energy, entropy, mean conductance, slope, and dynamic range; and the spectral descriptors include dominant frequency, spectral power, and band-power measures. Index- or identifier-like columns are explicitly detected and removed so that the model learns from physiological measurements rather than sample position. Each feature is min–max normalized to [0,1],

$${\tilde{x}}_{d}=\frac{x_{\mathrm{raw},d}-\min_{d}}{\max_{d}-\min_{d}},\;d=1,\ldots ,D,$$ (1)

which places all features within a common range, making subsequent sinusoidal encoding well-conditioned.

**Figure 2:**
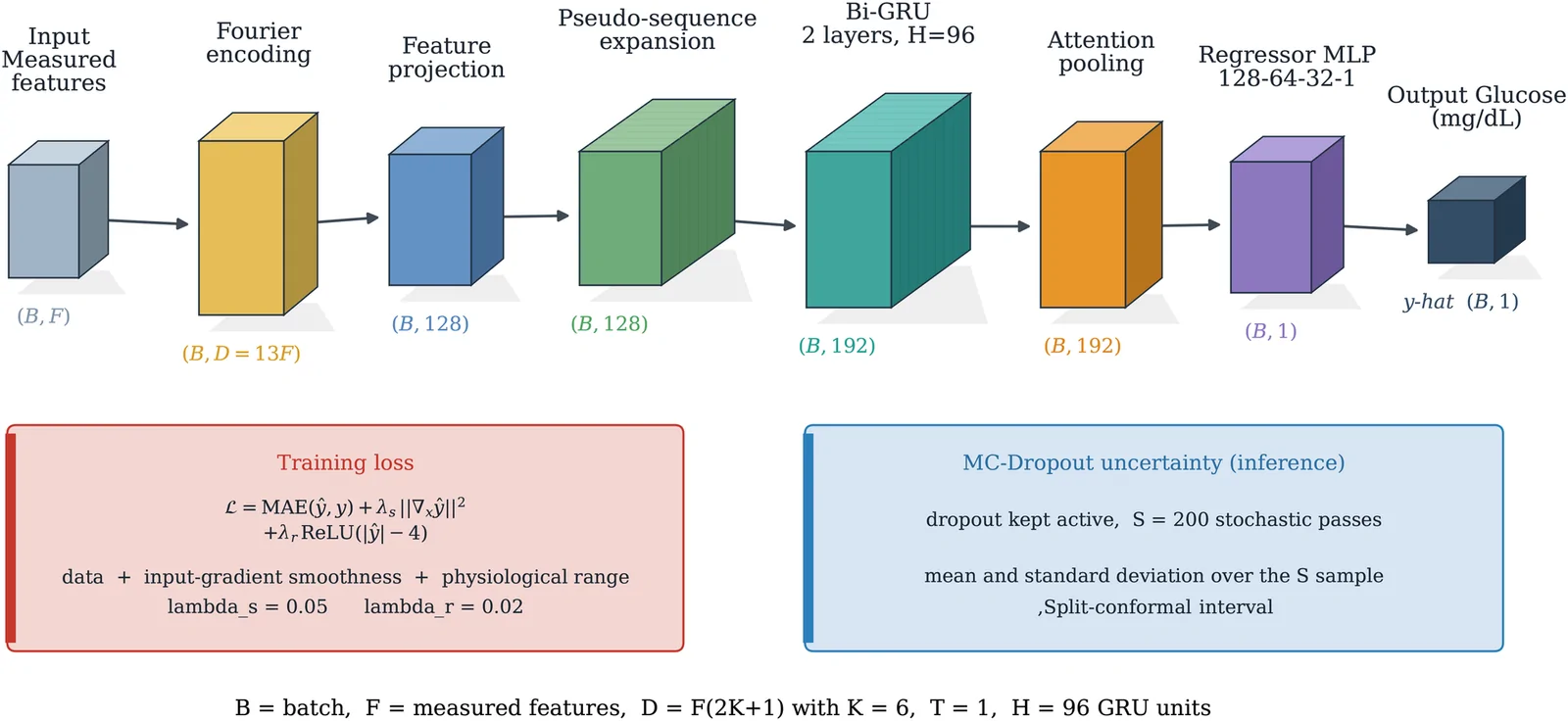
Architecture of the proposed PI-Uncertainty Fourier-BiGRU-NN. Fourier-encoded multimodal features are projected, mixed by an attentive bidirectional GRU, and regressed to glucose; MC-dropout is used to estimate predictive uncertainty.

### 2.2 Deterministic harmonic fourier feature encoding

To expose the high-frequency structure of the sensor-to-glucose mapping, each normalized feature is lifted by a deterministic harmonic Fourier positional encoding (34). For K frequencies, the encoding of the normalized vector $\tilde{x}$ is

$$\gamma (\tilde{x})=\left[\;\tilde{x},\;{\left\{\sin\;(2\pi k\tilde{x}),\;\cos\;(2\pi k\tilde{x})\right\}}_{k=1}^{K}\right],$$ (2)

where the sinusoids are applied element-wise. The encoded representation has dimension

M=D(1+2K), (3)

with K=6 in this work. The encoded features are then standardized to zero mean and unit variance,

$$x=\frac{\gamma (\tilde{x})-\mu_{\gamma }}{\sigma_{\gamma }},$$ (4)

yielding the network input $x\in R^{M}$.

### 2.3 Dense projection and pseudo-sequence expansion

The input is projected to a 128-dimensional latent embedding through a normalized nonlinear layer,

$$h_{0}=\mathrm{Dropout}\left(\mathrm{SiLU}(\mathrm{LN}(W_{\;p}x+b_{\;p}))\right),$$ (5)

where LN is layer normalization, SiLU(u)=uσ(u), and σ is the logistic sigmoid. The embedding is tiled T=1 times to form a pseudo-sequence

$$H=[\;h_{0};\;h_{0};\;\ldots ;\;h_{0}\;]\in R^{T\times 128},$$ (6)

so that the bidirectional recurrence and attention act as a deep nonlinear feature mixer over the static feature embedding.

### 2.4 Bidirectional GRU encoder

Each pseudo-step $H_{t}$ is processed by a two-layer bidirectional gated recurrent unit (GRU) (33). For a single direction with hidden state $s_{t}\in R^{H}$ (H=96), the GRU updates are

$$r_{t}=\sigma (W_{r}H_{t}+U_{r}s_{t-1}+b_{r}),$$ (7)

$$z_{t}=\sigma (W_{z}H_{t}+U_{z}s_{t-1}+b_{z}),$$ (8)

$${\tilde{s}}_{t}=\tanh\left(W_{n}H_{t}+r_{t}⊙(U_{n}s_{t-1})+b_{n}\right),$$ (9)

$$s_{t}=(1-z_{t})⊙{\tilde{s}}_{t}+z_{t}⊙s_{t-1},$$ (10)

where ⊙ is the Hadamard product. The forward and backward states are concatenated,

$$o_{t}=[{\vec{s}}_{t};\;{\overset{\leftarrow }{s}}_{t}]\in R^{2H},$$ (11)

producing the sequence $\{o_{t}{\}}_{t=1}^{T}$.

### 2.5 Additive attention pooling

The recurrent outputs are aggregated by an additive (Bahdanau-style) attention mechanism that learns the relative importance of each pseudo-step,

$$e_{t}=w_{a}^{⊤}\tanh\;(W_{a}o_{t}+b_{a}),$$ (12)

$$\alpha_{t}=\frac{\exp\;(e_{t})}{\sum_{\tau =1}^{T}\exp\;(e_{\tau })},\;c=\sum_{t=1}^{T}\alpha_{t}\;o_{t},$$ (13)

where $c\in R^{2H}$ is the attention-pooled context vector.

### 2.6 Regression head

The context vector is mapped to a scalar standardized glucose value through a multilayer perceptron with layer normalization and SiLU activations,

$$\hat{y}={\mathrm{MLP}}_{128\to 64\to 32\to 1}(c),$$ (14)

and the physical glucose estimate is recovered by inverting the target standardization,

$$\hat{g}=\sigma_{y}\;\hat{y}+\mu_{y}.$$ (15)

### 2.7 Composite regularized loss

Training minimizes a composite objective that augments the data-fitting term with two regularization terms. With N training samples, the data term is the mean absolute error between the standardized prediction ${\hat{y}}_{i}$ and the standardized target $y_{i}$:

$$L_{\mathrm{data}}=\frac{1}{N}\sum_{i=1}^{N}\left|{\hat{y}}_{i}-y_{i}\right|.$$ (16)

An input-gradient smoothness prior penalizes excessive sensitivity of the prediction with respect to the encoded input features, encouraging a smooth and robust sensor-to-glucose map without assuming any ordering among samples:

$$L_{\mathrm{smooth}}=\frac{1}{N}\sum_{i=1}^{N}{\left\|\nabla_{x_{i}}{\hat{y}}_{i}\right\|}_{2}^{2}=\frac{1}{N}\sum_{i=1}^{N}{\left\|\frac{\partial {\hat{y}}_{i}}{\partial x_{i}}\right\|}_{2}^{2}.$$ (17)

A soft standardized-output range constraint discourages predicted z-scores from drifting outside the statistical plausibility band $|{\hat{y}}_{i}|>4$:

$$L_{\mathrm{range}}=\frac{1}{N}\sum_{i=1}^{N}\max\left(0,|{\hat{y}}_{i}|-4\right).$$ (18)

The total objective is

$$L_{\mathrm{total}}=L_{\mathrm{data}}+\lambda_{s}L_{\mathrm{smooth}}+\lambda_{r}L_{\mathrm{range}},$$ (19)

where $\lambda_{s}=0.05$ and $\lambda_{r}=0.02$. Because Equation 18 is applied to the standardized prediction ${\hat{y}}_{i}$, it is a statistical z-score constraint rather than a physiological glucose-range constraint. A true physiological range constraint would be expressed on the inverse-standardized glucose estimate $\hat{g}$ in mg/dL.

### 2.8 MC-dropout scale estimation and split-conformal calibration

At inference, dropout is kept active and $T_{\mathrm{mc}}=200$ stochastic forward passes are drawn (35). Denoting the glucose prediction from the j-th stochastic pass as ${\hat{g}}^{\left(j\right)}$, the MC predictive mean and standard deviation are

$$\mu_{\mathrm{MC}}=\frac{1}{T_{\mathrm{mc}}}\sum_{\;j=1}^{T_{\mathrm{mc}}}{\hat{g}}^{\left(j\right)}$$ (20)

$$\sigma_{\mathrm{MC}}=\sqrt{\frac{1}{T_{\mathrm{mc}}-1}\sum_{\;j=1}^{T_{\mathrm{mc}}}{\left({\hat{g}}^{\left(j\right)}-\mu_{\mathrm{MC}}\right)}^{2}}.$$ (21)

The uncalibrated Gaussian reference interval is then approximated as

$$\left[\mu_{\mathrm{MC}}-1.96\sigma_{\mathrm{MC}},\;\mu_{\mathrm{MC}}+1.96\sigma_{\mathrm{MC}}\right].$$ (22)

At inference, dropout remains active and 200 stochastic forward passes are used to compute the MC-dropout predictive mean and standard deviation. We use these quantities as the prediction center and an input-dependent scale, respectively; we do not treat the uncalibrated Gaussian interval as the final prediction interval. For each experimental split, 10% of the training partition is reserved before fitting as a calibration subset. Model parameters, preprocessing statistics, and checkpoint selection use only the remaining proper-training subset. The test partition is not used for fitting or calibration. For each calibration observation i, define the normalized nonconformity score

$$r_{i}=\frac{\left|g_{i}-\mu_{\mathrm{MC}}(x_{i})\right|}{\max\left(\sigma_{\mathrm{MC}}(x_{i}),\varepsilon \right)},$$ (23)

where ε is a small positive constant used only to avoid division by zero.

For nominal coverage 1−α=0.95, let $n_{\mathrm{cal}}$ be the number of calibration observations and let $r_{\left(k\right)}$ denote the kth ordered calibration score. We use the finite-sample split-conformal quantile

$$k=\left\lceil (n_{\mathrm{cal}}+1)(1-\alpha )\right\rceil ,\;\hat{q}=r_{\left(k\right)},\;\alpha =0.05.$$ (24)

For a test input x, the calibrated prediction interval is

$$C_{0.95}(x)=\left[\mu_{\mathrm{MC}}(x)-\hat{q}\;s(x),\;\mu_{\mathrm{MC}}(x)+\hat{q}\;s(x)\right],\;s(x)=\max\left(\sigma_{\mathrm{MC}}(x),\varepsilon \right).$$ (25)

For LOSO fold j, the held-out participant contributes no records to proper training or calibration. Empirical coverage is the proportion of test labels contained in the interval, and mean interval width is the average upper-minus-lower endpoint difference. Split-conformal coverage relies on exchangeability between calibration and test observations. Because an unseen participant can introduce distribution shift and within-participant dependence, we report empirical LOSO coverage without claiming that the nominal 95% guarantee holds under this shift (36).

### 2.9 Evaluation metrics

Performance is reported with mean absolute error (MAE), root-mean-square error (RMSE), and the coefficient of determination ($R^{2}$):

$$\mathrm{MAE}=\frac{1}{N}\sum_{i=1}^{N}|{\hat{g}}_{i}-g_{i}|,$$ (26)

$$\mathrm{RMSE}=\sqrt{\frac{1}{N}\sum_{i=1}^{N}({\hat{g}}_{i}-g_{i}{)}^{2}},$$ (27)

$$R^{2}=1-\frac{\sum_{i=1}^{N}({\hat{g}}_{i}-g_{i}{)}^{2}}{\sum_{i=1}^{N}(g_{i}-\bar{g}{)}^{2}}.$$ (28)

### 2.10 Training and inference algorithm

Algorithm 1 summarizes the complete procedure. The data are stratified by glucose decile and split 80/20; features and target are standardized using statistics fit on the training split; the network is optimized with AdamW and a plateau learning-rate scheduler; and the best-loss checkpoint is retained for MC-dropout inference.

Algorithm 1 **PI-Uncertainty Fourier-BiGRU-NN training and inference**

**Require:** Features $X_{\mathrm{raw}}$, glucose targets g, frequencies K, epochs E, MC samples $T_{\mathrm{mc}}$

1: Remove index/identifier-like columns from $X_{\mathrm{raw}}$

2: $\tilde{X}\leftarrow$ min–max normalize $X_{\mathrm{raw}}$ ▹ Equation (1)

3: X← Fourier encode $\gamma (\tilde{X})$, then standardize ▹ Equation (2)–(4)

4: Stratified split into train/test; reserve 10% for calibration.

5: **for** e=1 to *E* **do**

6: Set x← trainable input; forward pass $\hat{y}=f_{\theta }(x)$

7: Compute $L_{\mathrm{data}},L_{\mathrm{smooth}},L_{\mathrm{range}}$ ▹ Equation (16)–(18)

8: $L_{\mathrm{total}}\leftarrow L_{\mathrm{data}}+\lambda_{s}L_{\mathrm{smooth}}+\lambda_{r}L_{\mathrm{range}}$

9: Update θ with AdamW; step LR scheduler on $L_{\mathrm{total}}$

10: Cache $\theta^{⋆}$ if $L_{\mathrm{total}}$ improves

11: **end for**

12: Restore the best parameters selected using proper-training data only.

13: On the calibration subset, compute the MC-dropout mean and scale from 200 stochastic passes and calculate normalized nonconformity scores.

14: Compute the finite-sample 0.95 conformal quantile from the calibration scores. ▹ Equation (24)

15: On the untouched test partition, return the MC-dropout mean and the calibrated 95% conformal interval. ▹ Equation (25)

## 3 Experimental results and discussion

The PhysioCGM dataset comprises 10 ambulatory participants with clinically diagnosed type 1 diabetes mellitus (T1DM). The cohort included participants aged 24–41 years (six male and four female), with body mass index values of 21.8–34.1 kg/m^2^ and HbA1c values of 5.4%–6.9%. All participants used a Dexcom G6 continuous glucose monitor (CGM) and an insulin pump as part of their routine diabetes management; however, participant-level medication regimens, insulin doses, and additional treatment information were not released (12). The multimodal dataset contains synchronized PPG and GSR measurements paired with reference blood glucose values. The dataset and acquisition details are summarized in Table 1. For each record, PPG- and GSR-derived features were extracted and concatenated into a single multimodal feature vector; the representative feature groups are listed in Table 2. The Empatica E4 is worn on the wrist and captures PPG at 64 Hz and EDA at 4 Hz. The ECG (250 Hz) comes from the separate Zephyr Bioharness chest strap, and ground-truth glucose comes from the Dexcom G6, which provides a subcutaneous glucose reading every 5 min (0.0033 Hz).

**Table 1:** Dataset and acquisition and preparation summary.

Item	Description
Number of participants	10 participants.
Recording duration	Approximately 10 min per participant.
Native PPG sampling frequency	64 Hz (Empatica E4).
Native GSR/EDA sampling frequency	4 Hz (Empatica E4).
Processed PPG sampling frequency	50 Hz after downsampling.
Processed GSR representation	GSR was up sampled using 50 Hz.
Total processed records	3,348 records with One glucose_mgdL value per record.
Total waveform values per record	3,200 values: 1,600 PPG values plus 1,600 GSR values.
Processed window duration	1,600 samples
Signal–label pairing rule	One 32 s PPG/GSR window centered on each Dexcom timestamp
Glucose range	Approximately 40–210 mg/dL (Dexcom each 5 min)
Feature groups	Morphological, statistical, and spectral descriptors
Primary split	Stratified 80/20 split by glucose decile
Additional validation	10-fold subject-independent split

**Table 2:** Representative input feature groups.

Group	Examples
PPG morphology	Peak amplitude, pulse interval, rise time, fall time, pulse width, area-related descriptors
PPG statistics	Mean, variance, standard deviation, skewness, kurtosis, energy, entropy
PPG spectral	Dominant frequency, spectral power, band-power descriptors
GSR statistics	Mean conductance, slope, variance, standard deviation, dynamic range
GSR temporal	Local changes, response magnitude, recovery-related descriptors

Dexcom G6 provided one glucose reference measurement every 5 min. For each valid Dexcom timestamp $t_{i}$, one 32 s physiological window was retained, defined as $W_{i}=[t_{i}-16\;s,\;t_{i}+16\;s)$. We assigned the Dexcom value $g_{i}$ recorded at the central timestamp $t_{i}$ directly as the label for $W_{i}$. We discarded physiological signals outside the retained windows.

The raw PPG signal is preprocessed using the “ppg_clean” function from NeuroKit2, which applies a second-order Butterworth bandpass filter (0.5–8 Hz) to remove baseline wander and high-frequency noise while preserving the physiological morphology of the PPG waveform. For EDA, the quality-assessment procedure used a low-pass FIR “de-spiking” filter, with the cutoff adjusted to 0.04 Hz to reflect the Empatica E4 EDA channel’s lower 4 Hz sampling rate.

Representative PPG and GSR physiological signals used for non-invasive glucose estimation are shown in Figure 3. The PPG trace contains periodic pulsatile waveform variations associated with blood-volume changes, whereas the GSR trace contains slower skin-conductance variations associated with autonomic activity. The concatenated PPG–GSR representation combines both modalities for multimodal analysis, allowing the model to exploit complementary cardiovascular and electrodermal information. The model was implemented in PyTorch and optimized with AdamW (learning rate $10^{-3}$, weight decay $10^{-5}$) for E=10,000 epochs using a ReduceLROnPlateau scheduler (patience 400, factor 0.5). The dropout rate was 0.12, the Bi-GRU used 2 layers with hidden size 96, the Fourier encoding used K=6 frequencies, and MC-dropout used $T_{\mathrm{mc}}=200$ samples, as summarized in Table 3. The primary evaluation used a sample-wise stratified 80/20 train–test split over glucose deciles. Because the primary split is sample-wise rather than subject-independent, samples from the same participant may appear in both training and test sets; the dedicated subject-independent evaluation below is therefore the more stringent and clinically relevant generalization check, and the gap between the two should be read together rather than the sample-wise split alone.

**Figure 3:**
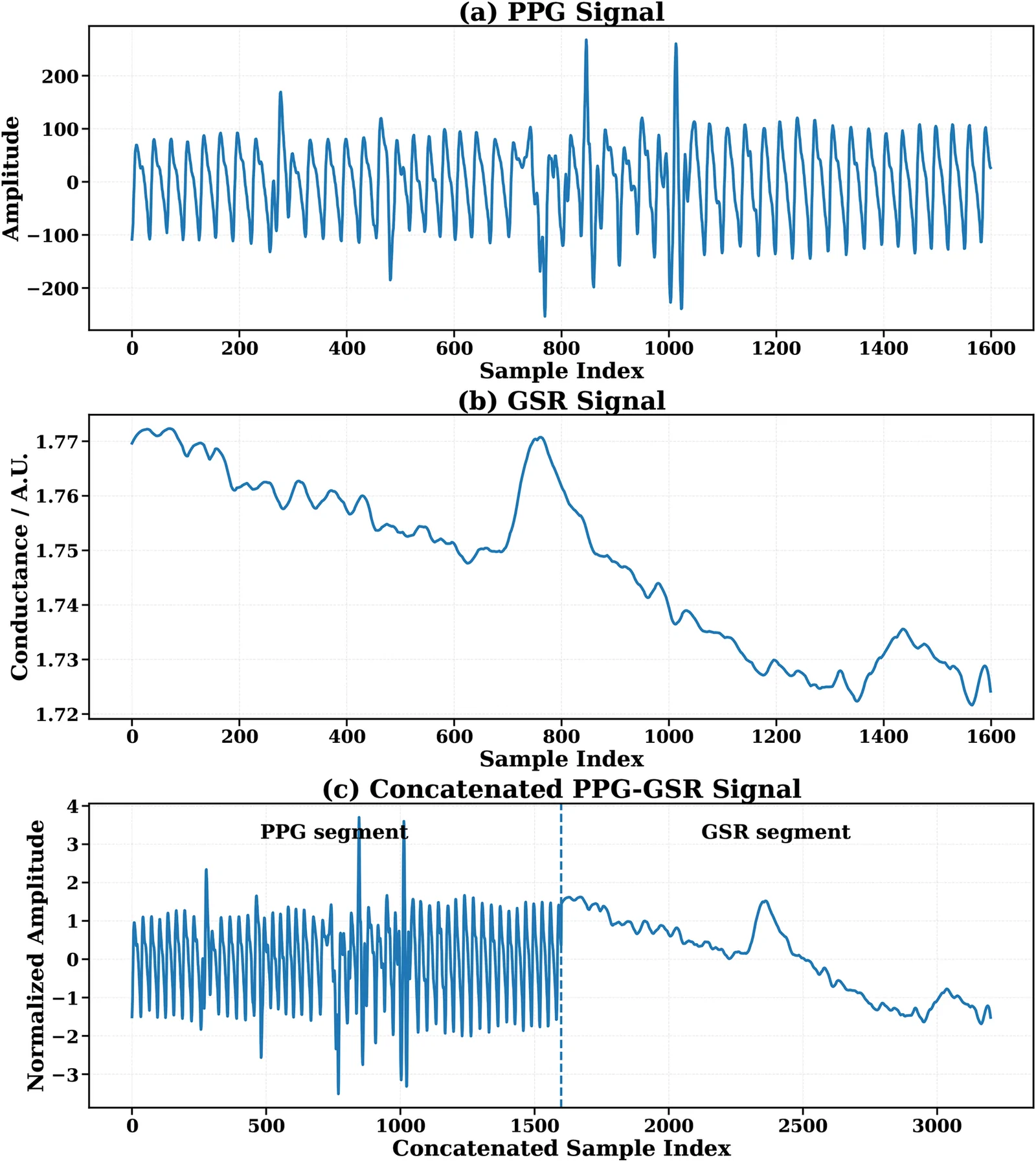
Representative physiological signals: **(a)** PPG signal, **(b)** GSR signal, and **(c)** concatenated PPG–GSR signal used for multimodal glucose prediction.

**Table 3:** Model and training hyperparameters.

Parameter	Value
Fourier frequencies K	6
Bi-GRU hidden size H	96
Bi-GRU layers	2
Pseudo-sequence length T	1
Dropout rate	0.12
MC-dropout samples $T_{\mathrm{mc}}$	200
$\lambda_{s}$ (smoothness)	0.05
$\lambda_{r}$ (range)	0.02
Optimizer / LR	AdamW / $10^{-3}$
Epochs	10,000

### 3.1 Training convergence

Figure 4 illustrates the training loss evolution of the proposed PI-Uncertainty Fourier-BiGRU-NN model over 10,000 epochs using a logarithmic loss scale. The total loss is defined as expressed in Equation 19. At the beginning of training, the total loss is relatively high, indicating that the model initially has a mismatch between the predicted and reference glucose values. During the first few thousand epochs, both the total loss and the data loss decrease rapidly, showing that the Fourier-encoded Bi-GRU network quickly learns the dominant non-linear relationship between the multimodal PPG–GSR features and blood glucose concentration. After approximately 3000–4000 epochs, the decrease becomes gradual, and the curves approach a stable plateau near $1.3\times 10^{-1}$, suggesting convergence of the main data-fitting objective.

**Figure 4:**
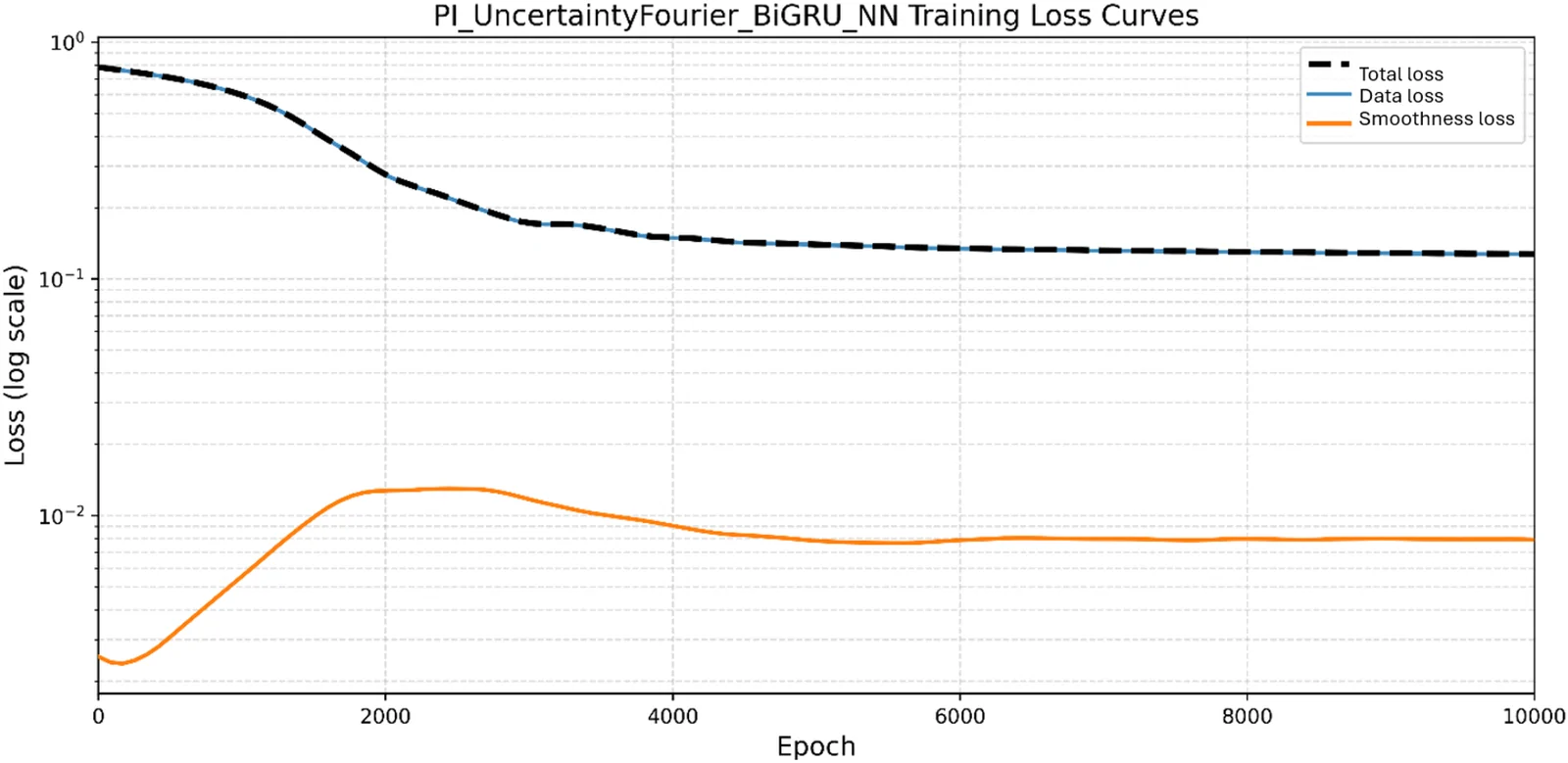
Training loss curves of the proposed method.

The smoothness loss remains much smaller than the data loss throughout training, with values on the order of $10^{-3}$ to $10^{-2}$. A temporary increase in the smoothness loss is observed around 1500–2500 epochs, after which it decreases and stabilizes. This behaviour indicates that the regularization term briefly constrains the model while the network adjusts its internal representation, but it does not dominate the optimization process. The close overlap between the total loss and data loss further confirms that the total objective is primarily governed by the data-fidelity term, while the smoothness component acts as a stabilizing auxiliary constraint. Overall, the smooth reduction of the loss curves and the absence of divergence demonstrate stable training of the proposed model. The final plateau indicates that the network has reached a steady optimum, where further epochs provide only minor refinement. This supports the effectiveness of the proposed uncertainty-aware Fourier Bi-GRU architecture for learning a robust sensor-to-glucose mapping from multimodal features.

Table 4 reports the headline metrics for the stratified held-out test split. The proposed model attains an MAE of 3.09±0.9110 mg/dL, an RMSE of 4.72 mg/dL, and $R^{2}=0.960$, indicating that it explains 96% of the variance in glucose under the evaluated split with a low average prediction error. The ratio RMSE/MAE≈1.53 suggests that the error distribution is dominated by small residuals rather than a large number of extreme outliers.

**Table 4:** Performance of the proposed methodology.

Model	MAE	RMSE	$R^{2}$	Mean $\sigma_{\mathrm{MC}}$
	(mg/dL)	(mg/dL)		(mg/dL)
Proposed methodology	3.09±0.9110	4.72	0.960	≈1.0

For the corresponding leakage-controlled sample-wise analysis, the final nominal 95% split-conformal prediction intervals achieved an empirical coverage of 95.66% with a mean interval width of 14.71 mg/dL.

Table 5 summarizes preliminary agreement metrics: the MARD is approximately 3%, roughly 88% of predictions fall within ±7 mg/dL, and about 93% fall within ±10 mg/dL. These figures indicate encouraging agreement on the sample-wise split and are corroborated by the Clarke Error Grid analysis in Section 3.3. Figure 5 plots predicted against actual glucose: the points cluster tightly around the ideal $45^{\circ }$ line, the fitted prediction trend is nearly coincident with it, and most samples lie inside the ±10 mg/dL band across the evaluated glucose range.

**Table 5:** Preliminary agreement metrics of the proposed model.

Criterion	Value
Mean absolute relative difference (MARD)	≈3%
Predictions within ±5 mg/dL	≈80%
Predictions within ±7 mg/dL	≈88%
Predictions within ±10 mg/dL	≈93%
95% limits of agreement	≈±9 mg/dL

**Figure 5:**
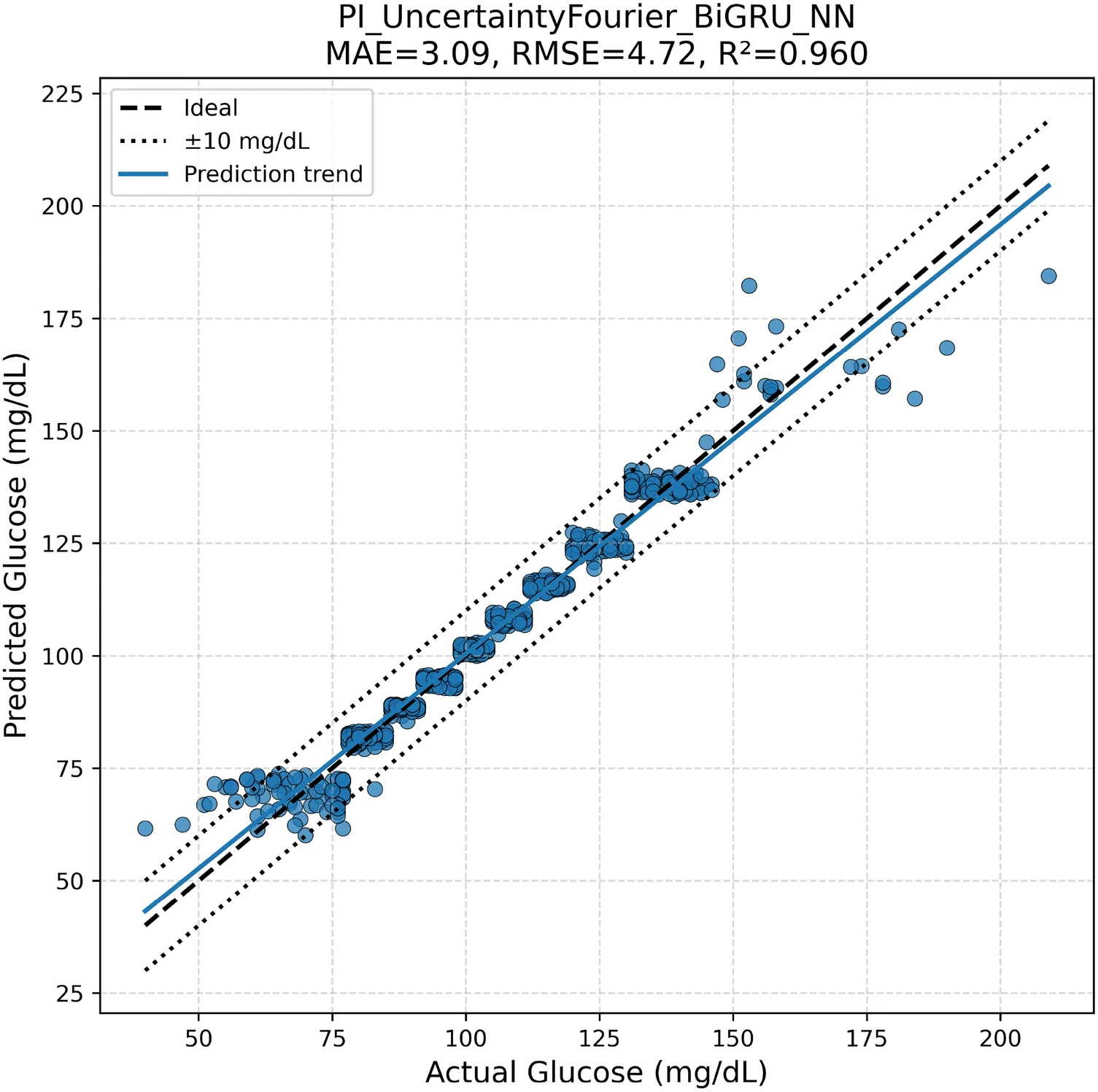
Predicted vs. actual glucose. The dashed line is the ideal identity, dotted lines mark the ±10 mg/dL band, and the solid line is the fitted prediction trend.

### 3.2 Error distribution and clinical bands

Figure 6 shows the absolute-error distribution, which is sharply concentrated near zero with a mean of 3.09±0.9110 mg/dL and a long but sparse tail. Figure 7 relates absolute error to the actual glucose value: errors remain small and homoscedastic across the densely sampled normoglycemic range (∼75–145 mg/dL) and grow only at the sparsely populated low (<60 mg/dL) and high (>150 mg/dL) extremes, where fewer training examples are available. The majority of predictions fall below the 7 mg/dL and 10 mg/dL reference lines, supporting the preliminary agreement trends in Table 5.

**Figure 6:**
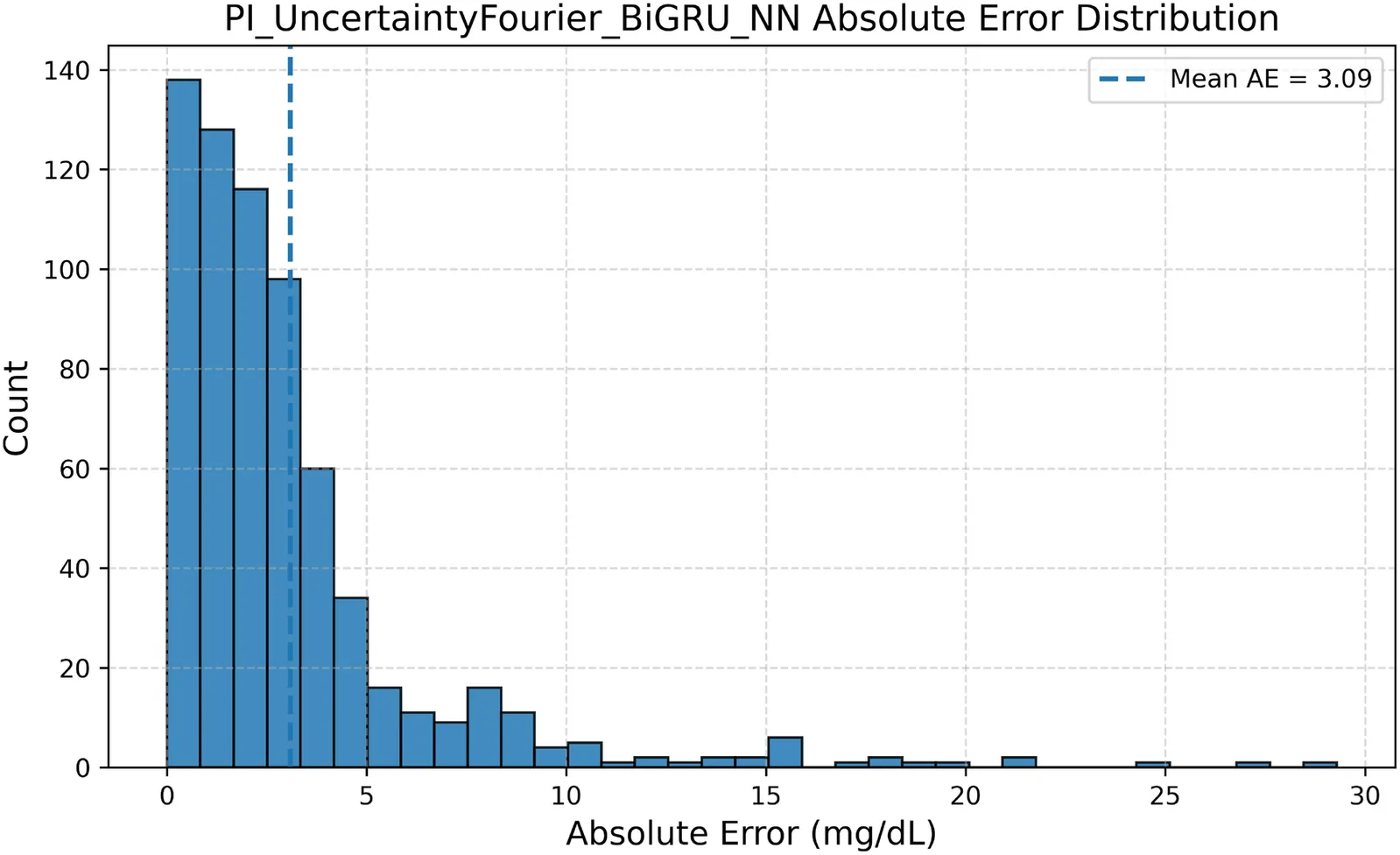
Absolute-error distribution on the test set; the dashed line marks the mean absolute error.

**Figure 7:**
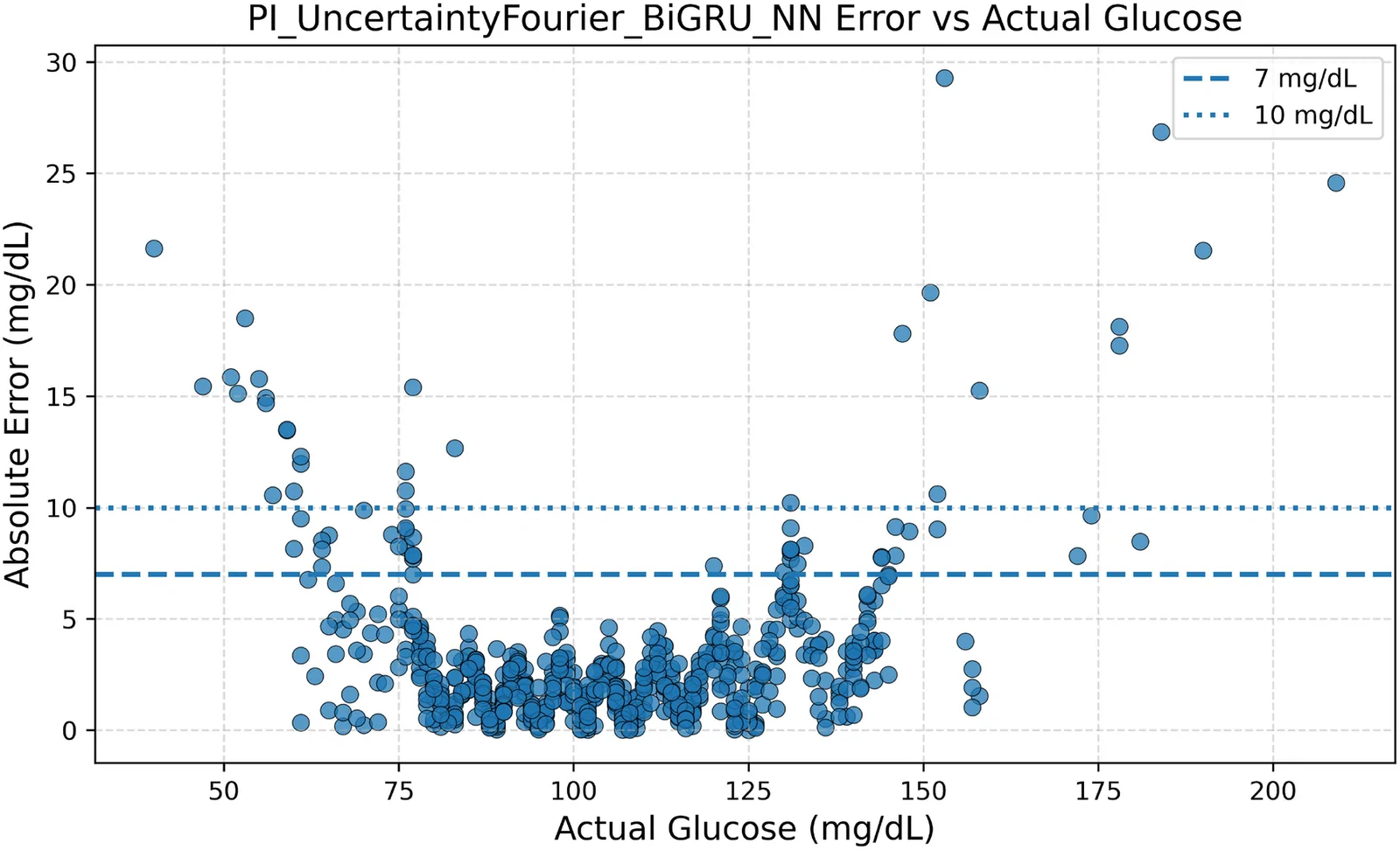
Absolute error vs. actual glucose. Errors are smallest in the densely sampled mid-range and increase at the data-sparse extremes.

The Bland–Altman plot in Figure 8 assesses agreement between predicted and reference glucose. The mean bias is close to zero, indicating no systematic over- or under-estimation, and almost all differences lie within the ±1.96 SD limits of agreement. The few points outside the limits again correspond to the glycemic extremes, mirroring the error-vs.-actual trend. Figure 9 overlays predicted and actual glucose for the test samples ordered by reference value. The prediction closely follows the actual profile across all glycemic levels, including the transitions between low, normal, and elevated regions, showing that the attentive Bi-GRU captures the nonlinear sensor-to-glucose mapping rather than regressing toward the mean. Table 6 compares the proposed model with same-dataset baseline regressors, including SVR, Random Forest, XGBoost, MLP, and a Bi-GRU variant without Fourier encoding. All models use 80/20 train–test partition, random seed 42, 10000-epoch budget, full-batch training, AdamW optimizer, constant learning rate of 0.001, weight decay of $10^{-5}$ and 200 Monte Carlo dropout samples at evaluation. Input and target standardization are fitted on the training partition only and then applied unchanged to the held-out test partition. For the Fourier-input comparison, every neural architecture receives the same 33 train-standardized Fourier features. The proposed Fourier Bi-GRU contains 349,698 trainable parameters (96 hidden units, two Bi-GRU layers, dropout = 0.12). The other baseline methods are parameter-matched to this number. All use the same MAE data-loss term plus smoothness, and range terms, with coefficients of 1, 0.05, and 0.02, respectively.

**Figure 8:**
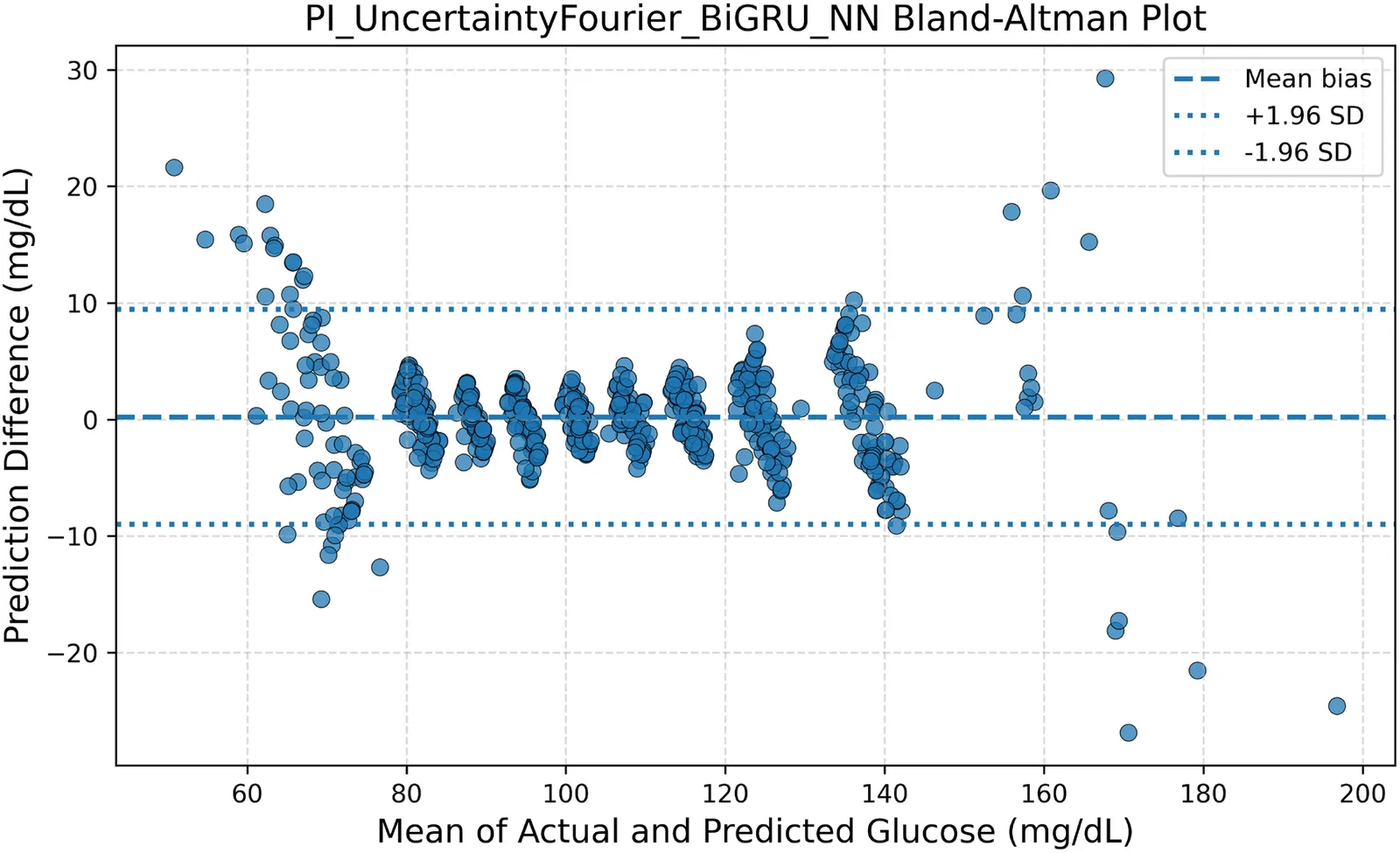
Bland–Altman agreement plot. Mean bias is near zero and the limits of agreement are narrow.

**Figure 9:**
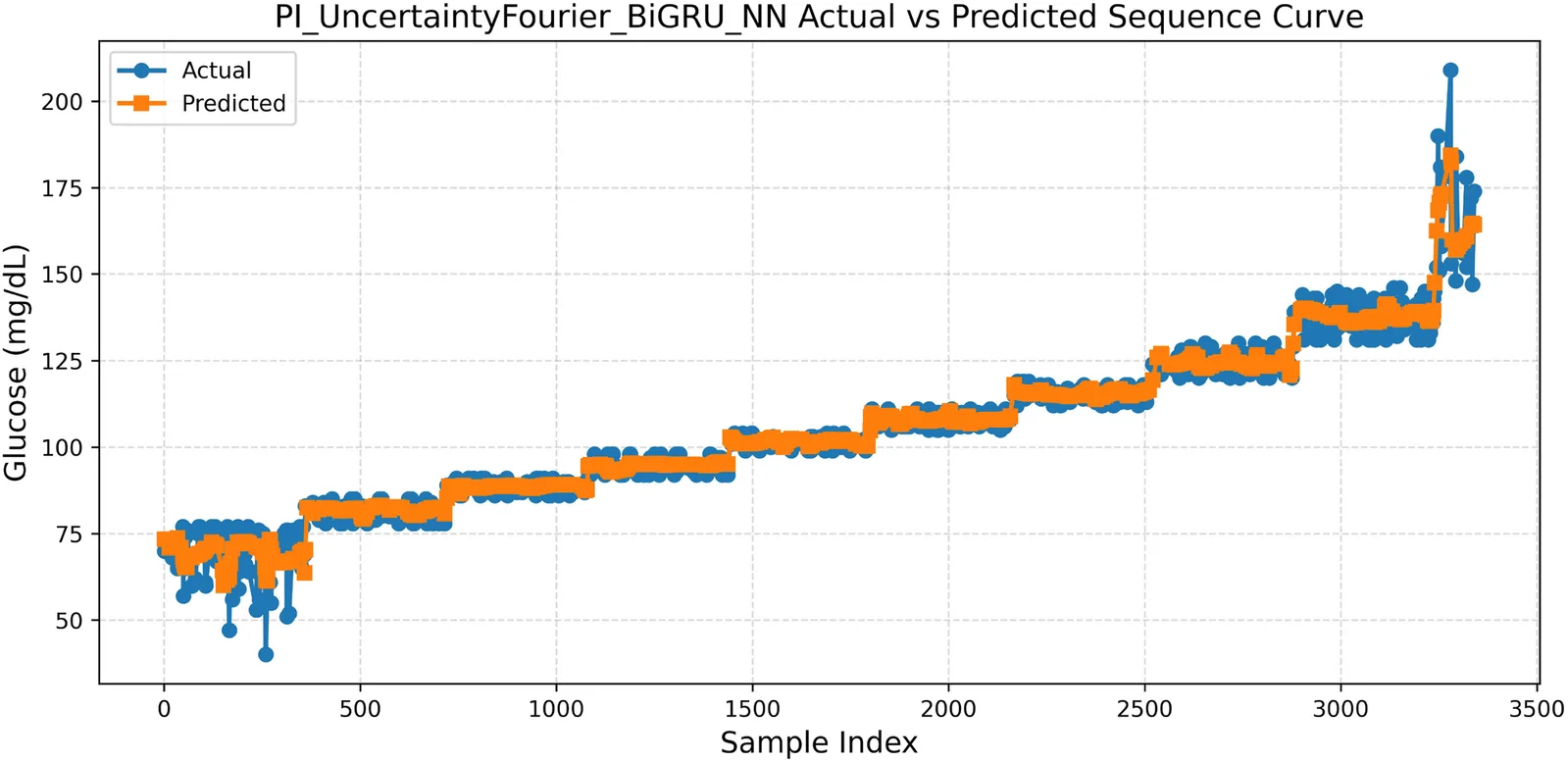
Actual vs. predicted glucose for ordered test samples; the prediction tracks the reference across the full range.

**Table 6:** Same-dataset baseline model comparison.

Model	MAE	RMSE	$R^{2}$
	(mg/dL)	(mg/dL)	
SVR	10.32±0.3365	14.68	0.742
Random Forest	8.91±1.0256	12.74	0.791
XGBoost	7.46±0.3658	10.83	0.834
MLP	6.18±0.6584	8.94	0.879
Bi-GRU without Fourier	4.18±1.0258	6.21	0.928
Proposed	3.09±0.9110	4.72	0.960

### 3.3 Clarke error grid analysis of the proposed model

To assess clinical safety beyond aggregate error metrics, we evaluated the estimator using the Clarke Error Grid in Figure 10, which classifies each prediction according to its clinical consequence. On the stratified held-out split, the model placed 96.3% of test predictions in Zone A and 2.8% in Zone B (benign deviation that would not alter treatment), yielding 99.1% in the combined A+B region that is considered clinically acceptable. No predictions fell in Zone C (unnecessary correction) or Zone E (erroneous opposite treatment), and only 0.9% fell in Zone D. The Zone D points coincide with the data-sparse hypo- and hyper-glycemic extremes already identified in the error analysis, consistent with the class imbalance of the normoglycemic-dominated dataset. This grid was computed on the sample-wise split; the subject-independent split in Section 3.5 has not yet been assessed on the Clarke grid and is identified as future work below.

**Figure 10:**
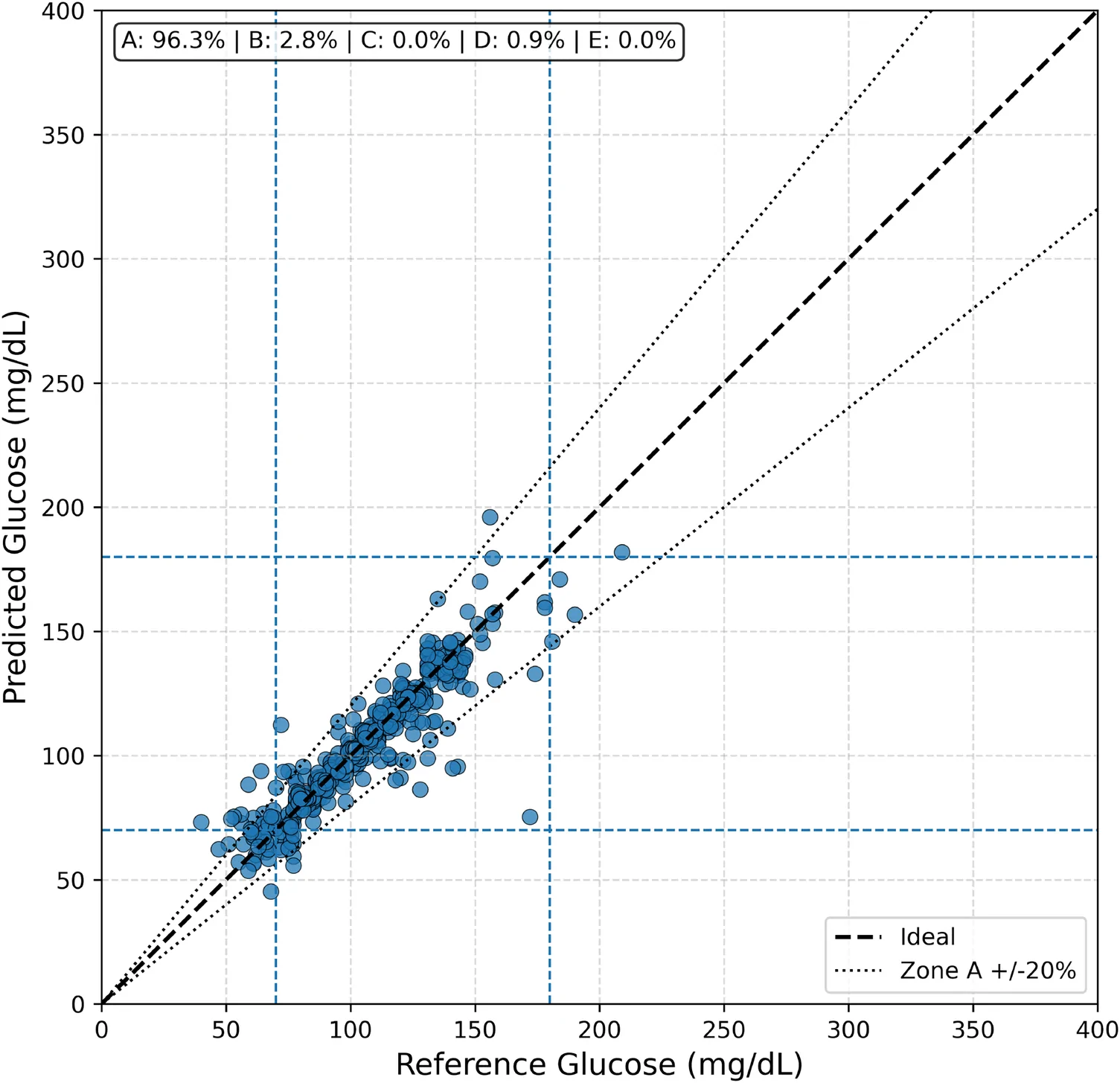
Clarke error grid analysis of the proposed model.

### 3.4 Feature attribution using permutation importance

To interpret which parts of the multimodal window the network relies on, we computed permutation importance, defined as the increase in test MAE when the values of a given input sample are randomly shuffled. Figures 11, 12 show the twenty most influential PPG and GSR samples, respectively. Two observations follow. First, within each modality the importance is distributed across many samples rather than concentrated in a single point, reflecting the high temporal correlation of adjacent samples in the 1,600-point window: permuting one sample only modestly degrades performance because the recurrent encoder recovers the information from neighbouring samples. The informative quantity is therefore the relative ranking and the cross-modality scale, rather than the absolute per-sample values. Second, the peak PPG importance (≈0.063 mg/dL) exceeds the peak GSR importance (≈0.0065 mg/dL) by roughly an order of magnitude, indicating that PPG is the dominant predictor while GSR supplies a weaker but complementary electrodermal signal.

**Figure 11:**
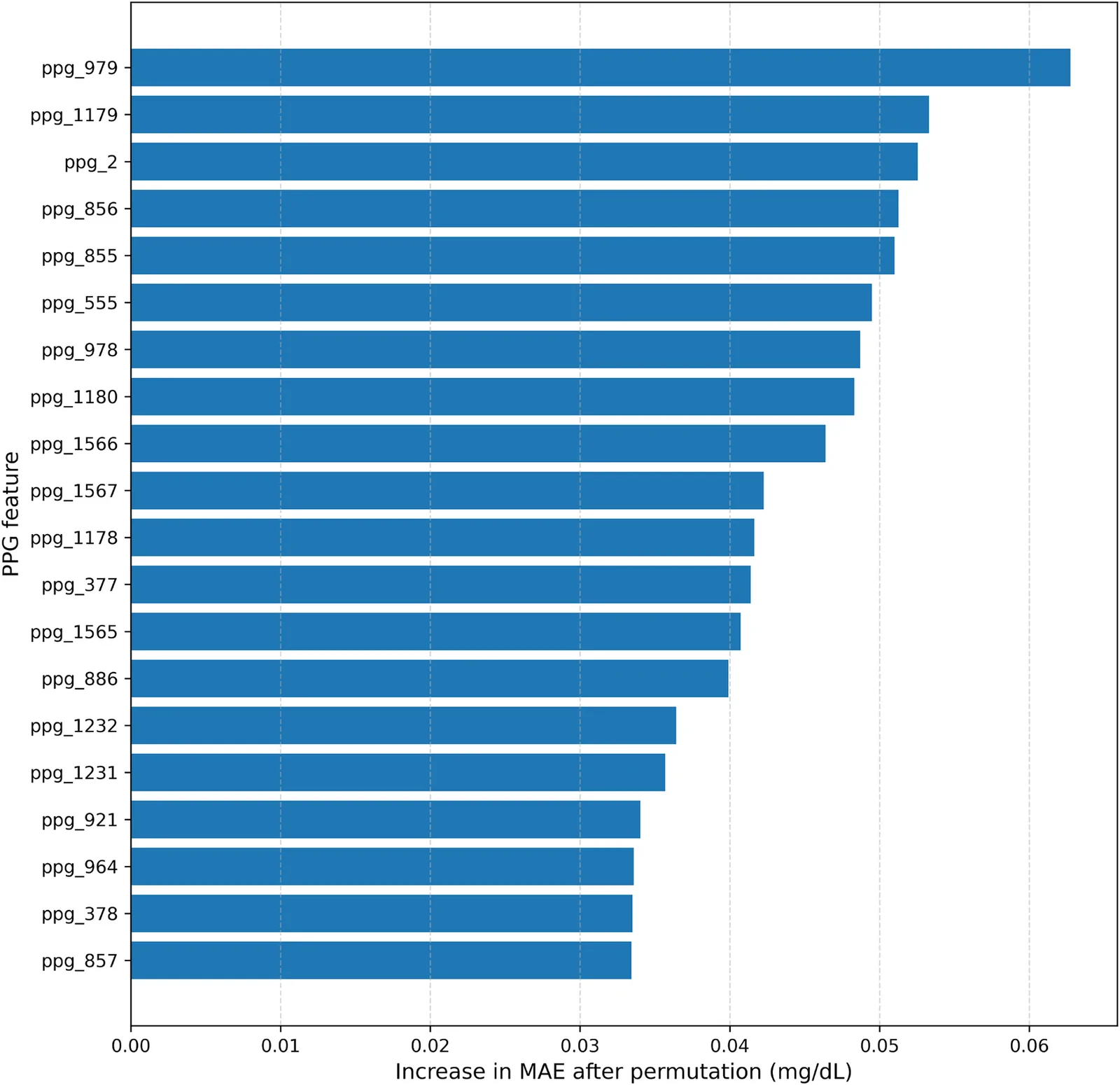
PPG permutation importance (top-20 samples).

**Figure 12:**
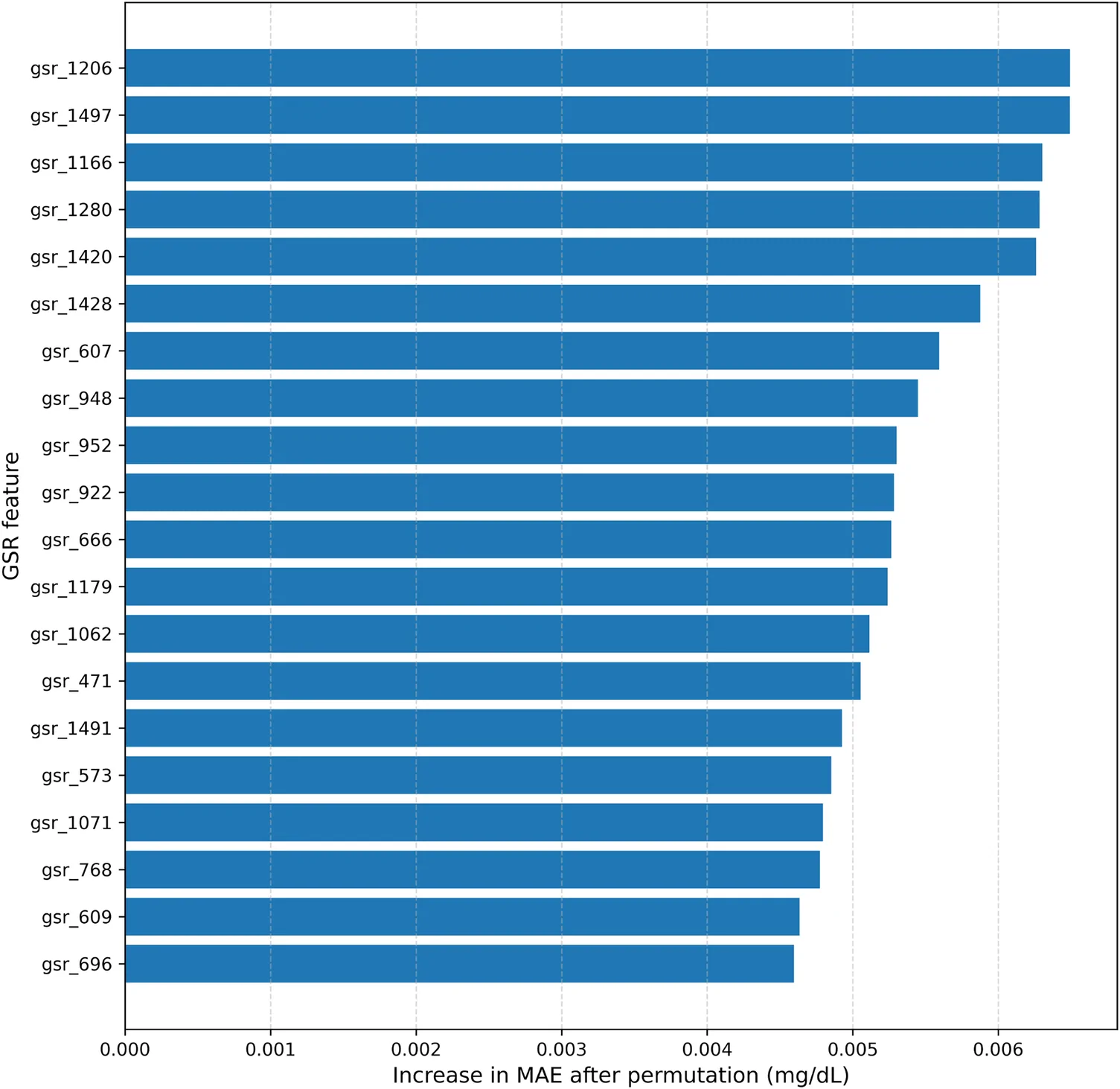
GSR permutation importance (top-20 samples).

### 3.5 Subject-independent generalization evaluation

To further assess the generalization capability of the proposed PI-Uncertainty Fourier-BiGRU-NN model, a subject-independent validation experiment was performed. In this protocol, data from Patients 1–8 were used exclusively for model training, whereas Patients 9 and 10 were completely withheld during training and used only for testing. This evaluation is more stringent than a conventional random sample-wise split because the test data are obtained from unseen subjects. Therefore, the experiment evaluates whether the learned PPG–GSR-to-glucose mapping can transfer across inter-subject physiological variability rather than merely interpolate among samples from subjects already observed during training. Figure 13 shows the predicted glucose values against the reference glucose values for the subject-independent test split. The model achieved an MAE of 5.63 mg/dL, an RMSE of 8.88 mg/dL, and an $R^{2}$ value of 0.881 on the unseen Patients 9 and 10. This is a degradation of approximately 82% in MAE and 88% in RMSE relative to the stratified sample-wise split (Table 4), and a drop of 0.079 in $R^{2}$; the degradation is expected because the model is required to predict glucose values for subjects whose physiological characteristics were not available during training. Such inter-subject differences may include variations in vascular tone, skin conductance response, peripheral perfusion, sensor coupling, and individual glucose distribution.

**Figure 13:**
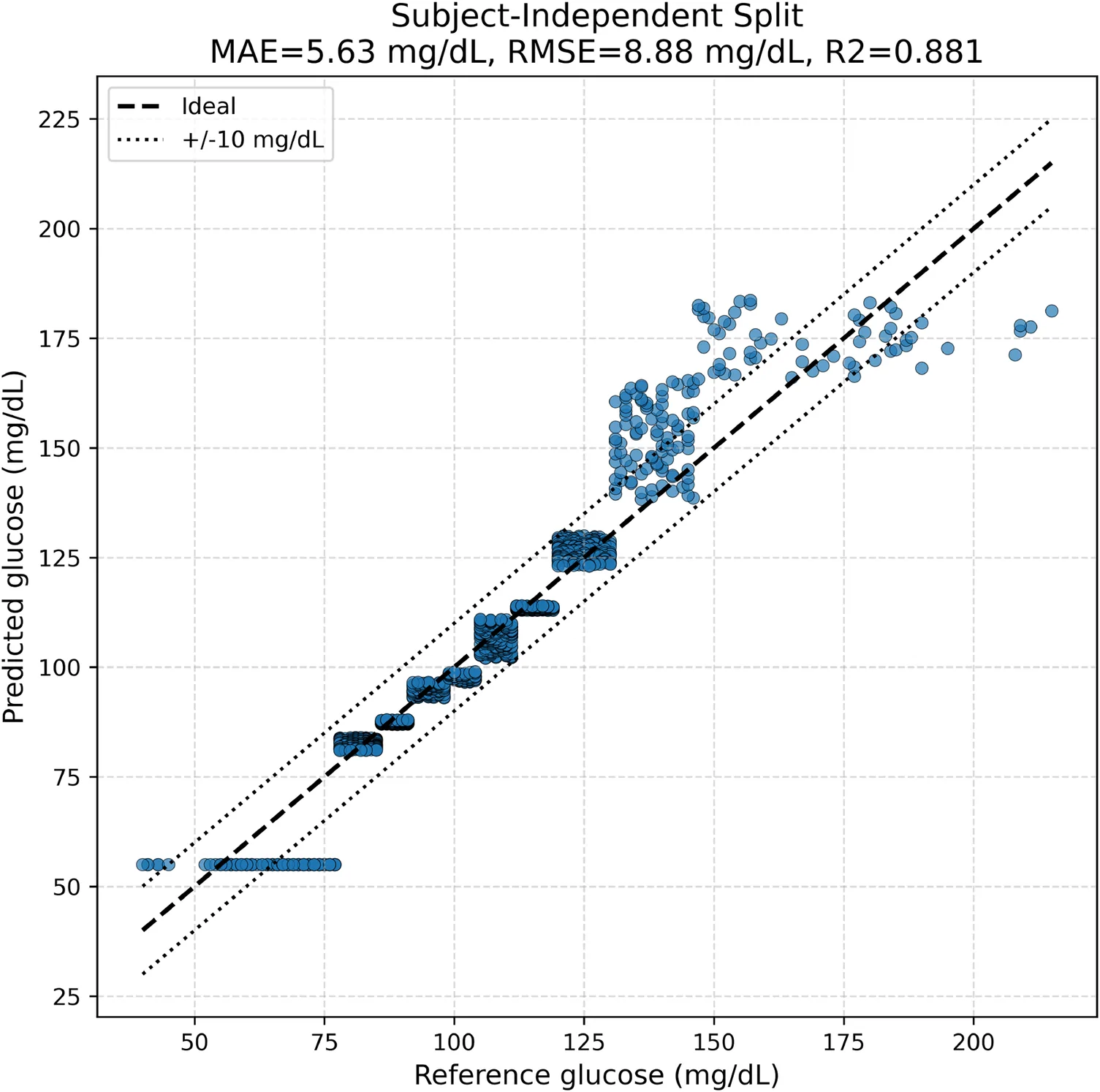
Subject-independent predicted vs. reference glucose values.

The absolute-error distribution for the subject-independent test split is shown in Figure 14. The error histogram is concentrated near low absolute-error values, with the mean absolute error marked at 5.63 mg/dL. A substantial number of predictions exhibit errors below approximately 5–7 mg/dL, while only a sparse right tail extends toward larger errors. These larger deviations are mainly attributed to the difficulty of extrapolating to unseen subject-specific physiological responses, especially in glucose regions where the target subjects exhibit different sensor–glucose relationships from the training subjects. Nevertheless, the strongly right-skewed distribution demonstrates that the proposed model maintains useful predictive accuracy under a strict unseen-subject testing scenario, while also confirming that subject-independent generalization is the dominant remaining source of error in the proposed pipeline, ahead of the glycemic-extreme effects discussed for the sample-wise split.

**Figure 14:**
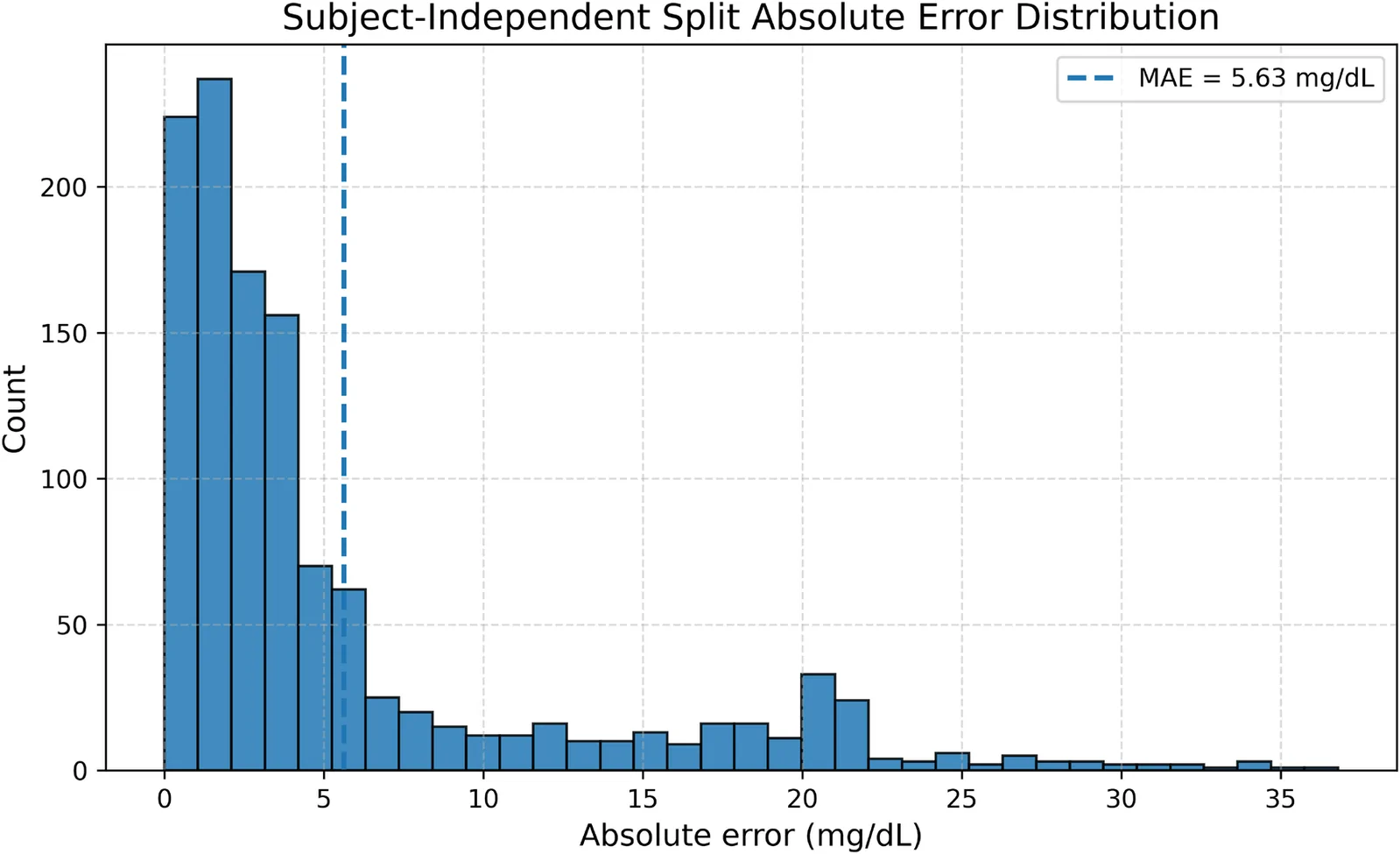
Absolute-error distribution for the subject-independent split.

### 3.6 Validation using LOSO analysis

Table 7 summarizes the 10-fold leave-one-subject-out evaluation of the proposed model. The MAE remained relatively stable across the 10 subject folds, ranging from 16.191 to 17.359 mg/dL, with a mean of 16.751±0.435 mg/dL.

**Table 7:** Ten-fold leave-one-subject-out (LOSO) performance of the proposed model.

Fold	Held-out target range	MAE
	(mg/dL)	(mg/dL)
1	43–215	17.278
2	40–211	16.888
3	53–209	16.494
4	41–205	16.326
5	55–183	16.297
6	40–212	17.014
7	40–208	16.524
8	43–206	17.359
9	51–195	16.191
10	43–209	17.143
Mean ± SD	–	16.751±0.435

Using the corresponding fold-specific calibration subsets, which contained records only from the nine training participants, the pooled out-of-fold nominal 95% split-conformal prediction intervals achieved an empirical coverage of 90.63% with a mean interval width of 51.48 mg/dL. The shortfall from the nominal 95% level is reported because the held-out participant may differ systematically from the participants represented in the proper-training and calibration subsets.

The held-out glucose ranges were broad in all folds, spanning approximately 40–215 mg/dL across the complete evaluation. The MAE for LOSO evaluation is higher than the sample-wise because the sample-wise uses closely related records that may occur in both the training and test sets. In contrast, the LOSO evaluation reduces local interpolation opportunities and exposes distributional differences between the subjects and the training data. The performance reduction additionally reflects inter-individual variation in physiology, sensor placement, skin properties, and glucose distribution. Therefore, the LOSO result provides a more conservative estimate of generalization than the sample-wise result.

Tables 8, 9 provide an additional, more demanding Leave-one-subject-out (LOSO) sensitivity analysis. Clarke Zone A agreement varied substantially across subjects, from 8.68% to 100.00%, while combined Zones A+B agreement ranged from 52.99% to 100.00%. The pooled glycemic-range analysis showed that performance was most stable in the target range of 70–180 mg/dL (n=3146), with a 99.49% Clarke A+B agreement. In contrast, performance was substantially poorer in the hypoglycemic range (<70 mg/dL; n=168; Clarke A+B =15.48%) and the hyperglycemic range (>180 mg/dL; n=26; Clarke A+B =100%). The apparently high Clarke A+B value in the hyperglycemic subgroup should be interpreted cautiously because of the very small sample size and the large numerical errors. Overall, these findings indicate that the proposed method requires further development and validation in hypoglycemic and hyperglycemic conditions. A future study with a larger cohort, and sufficient numbers of hypo- and hyperglycemic observations is required for formal subject-independent clinical validation.

**Table 8:** Clarke error grid results for the 10-fold LOSO evaluation.

Subjects	Zone A (%)	Zones A+B (%)
1	8.68	52.99
2	75.45	99.70
3	87.43	100.00
4	85.33	100.00
5	100.00	100.00
6	96.11	100.00
7	64.67	100.00
8	99.70	100.00
9	80.54	100.00
10	34.13	100.00

**Table 9:** Pooled glycemic-range performance for the 10-fold LOSO evaluation.

Glycemic range	n	Clarke A+B **(%)**
Hypoglycemic: <70 mg/dL	168	15.48
Target range: 70–180 mg/dL	3,146	99.49
Hyperglycemic: >180 mg/dL	26	100.00

## 4 Ablation study and discussion

The ablation experiments in Table 11 were designed to isolate the contribution of each model component while keeping the same preprocessing pipeline, optimizer, train–test split, training schedule, and MC-dropout inference procedure.

The results in Table 11 show that the high held-out split accuracy follows from matching the architecture and training objective to the problem’s structure. PPG encodes optical pulsatile-vascular correlates of glucose while GSR encodes sympathetic/electrodermal correlates, and the two fail under different conditions (PPG under motion/perfusion, GSR under thermal/affective drift). Their fusion provides redundant, mutually correcting evidence, which is the main reason the dense normoglycemic range is estimated with errors of only 1–3 mg/dL (Figure 7). Plain inputs bias networks toward low-frequency functions, under-fitting small but informative feature variations. Lifting each normalized feature through K=6 harmonics (Equation 2) supplies an explicit high-frequency basis, letting the regressor resolve fine differences between adjacent glycemic levels—visible in Figure 9, where the prediction tracks stepwise transitions instead of collapsing toward the mean. The bidirectional GRU with additive attention (Equations 11–13) serves as a deep gated feature mixer whose gating and attention weights learn non-linear, context-dependent interactions among the encoded features, thereby adding representational capacity in the transition regions without destabilizing training. The input-gradient smoothness prior (Equation 17) suppresses noise-driven output sensitivities and the soft standardized-output range constraint (Equation 18) keeps predicted z-scores statistically plausible, without imposing a clinical mg/dL range; together they reduce variance and overfitting, reflected in the near-zero Bland–Altman bias (Figure 8) and the tight, outlier-light error distribution (Figure 6). MC-dropout yields a predictive standard deviation that grows with error, so the few large errors at the extremes carry high uncertainty and can be flagged, thereby raising the system’s actionable accuracy above what the raw statistics suggest. The novelty of this work is unifying, within a single end-to-end estimator, four ingredients previously explored only in isolation for non-invasive glucose sensing. To our knowledge, deterministic harmonic Fourier positional encoding of fused PPG and GSR features has not been used for blood glucose regression. Prior PPG-only and GSR-only estimators operate on raw or hand-crafted features and therefore under-resolve the high-frequency feature–glucose structure; applying the encoding to the *measured* features (rather than a sample index) allows a compact network to reach $R^{2}=0.960$. Repurposing a bidirectional GRU with additive attention to mix a static multimodal feature vector—rather than to model a literal time series—provides recurrent gating capacity for tabular features while keeping the model small and trainable on modest data. The objective combines MAE fitting with an input-gradient smoothness prior and a soft constraint on a standardized output range. Unlike monotonicity priors used in some sequence models, the smoothness prior makes *no* assumption about sample ordering, so it applies to cross-sectional multimodal data while still enforcing a robust, smooth sensor-to-glucose mapping. The model reports per-sample empirical 95% prediction intervals whose widths tend to increase with error, thereby turning a black-box point estimator into a reliability-aware one. This combination distinguishes the method: each component addresses a specific failure mode (under-resolution, limited mixing capacity, over-fitting, and silent errors), and together they yield an accurate estimator on data drawn from previously observed subjects, with quantified but not yet fully characterized degradation on unseen subjects. Table 10 situates the proposed method among representative non-invasive approaches. Direct numerical comparison is confounded because reported studies use different cohorts, reference devices, and evaluation protocols; the table therefore contrasts modality, modeling strategy, and the headline outcome reported by each work. Single-modality PPG estimators based on hand-crafted features and classical learners established feasibility but typically report coarser accuracy or only categorical classification (13, 14), while clinically framed PPG regressors report agreement primarily through error-grid zones (17). Fusing PPG with auxiliary features improves accuracy (24), and recent attentive recurrent models advance continuous monitoring (32). Recent multimodal systems further fuse PPG with electrodermal activity, skin temperature, or ECG to improve robustness and accuracy (10, 12, 37). The proposed Fourier-encoded multimodal Bi-GRU with uncertainty reports an MAE of 3.09±0.9110 mg/dL, RMSE of 4.72 mg/dL, and $R^{2}=0.960$ on the held-out sample-wise split together with MC-dropout-scaled split-conformal prediction intervals, and an MAE of 16.75 mg/dL on the LOSO; reporting both is intended to give a more complete and honest picture than the sample-wise number alone. The ablation study in Table 11 evaluates the contribution of each major component of the proposed PI-Uncertainty Fourier Bi-GRU-NN model. The proposed full model achieved the best overall point-prediction performance, with an MAE of 3.09±0.9110 mg/dL, an RMSE of 4.72 mg/dL, and a coefficient of determination of $R^{2}=0.960$. The final calibrated prediction-interval results are reported separately for the sample-wise and LOSO evaluations. The PPG-only and GSR-only variants show considerably higher errors, with MAE values of 5.92 mg/dL and 7.14 mg/dL, respectively, confirming that multimodal fusion of PPG and GSR provides complementary features for glucose estimation. The poorer performance of the GSR-only model indicates that GSR alone is less discriminative for accurate glucose regression, although it still contributes useful electrodermal information when fused with PPG. Removing Fourier encoding increases the MAE from 3.09±0.9110 mg/dL to 4.18 mg/dL and reduces $R^{2}$ from 0.960 to 0.928, demonstrating that Fourier feature mapping improves the representation of nonlinear and high-frequency relationships between physiological signals and glucose concentration. The removal of the Bi-GRU encoder results in greater degradation, with MAE increasing to 5.36 mg/dL and $R^{2}$ decreasing to 0.889, indicating that gated recurrent feature mixing is important for capturing complex interactions in the encoded feature space. Similarly, removing attention pooling reduces performance, indicating that adaptive weighting of latent representations improves glucose prediction. The model trained without the composite regularized loss also performs worse than the full model, with MAE increasing to 4.47 mg/dL and RMSE increasing to 6.73 mg/dL, confirming that the smoothness and standardized-output range constraints improve prediction stability and generalization. Overall, the ablation results validate that the proposed architecture benefits from the joint integration of multimodal PPG–GSR fusion, Fourier feature encoding, attentive Bi-GRU learning, composite regularization, and MC-dropout uncertainty estimation, on the sample-wise evaluation protocol used throughout this study.

**Table 10:** Qualitative comparison with representative non-invasive methods.

Work	Modality	Model	Metrics
*Single-modality methods*			
(13)	Smartphone PPG	Gaussian feat. + GSVM	Acc. 81.49% (3-class); 97.54% valid-signal
(14)	Smartphone PPG	PLS regression	SEP 17.02 mg/dL
(17)	In-ear PPG	Ensemble reg. trees	∼82% Clarke zone A; RMSE 17.5–42.8 mg/dL
(32)	PPG/CGM	Bi-recurrent attention	RMSE 15.42, MAE 12.01 mg/dL (30 min)
*Multimodal methods*			
(24)	Wristband PPG + physio	XGBR	MAE 1.76, SEP 5.53 mg/dL, $R^{2}$ 0.995
(10)	Multi-sensor (PPG + aux.)	ML	Portable prototype; no MAE/RMSE reported
(37)	PPG + EDA + ST (+food)	Multi-stream cross-modal attn.	MAE 13.51, RMSE 17.26 mg/dL; 99.43% A + B
Proposed (sample-wise)	PPG + GSR from PhysioCGM	Fourier Bi-GRU+MC-dropout	MAE 3.09±0.9110, RMSE 4.72, $R^{2}$ 0.960
Proposed (LOSO)	PPG + GSR from PhysioCGM	Fourier Bi-GRU+MC-dropout	MAE 16.751±0.435

**Table 11:** Ablation study of the proposed PI-Uncertainty Fourier Bi-GRU-NN model.

Model variant	MAE	RMSE	$R^{2}$	Mean $\sigma_{\mathrm{MC}}$	Empirical 95% PI
	(mg/dL)	(mg/dL)		(mg/dL)	Coverage % (uncalibrated)
PPG-only input	5.92±0.2356	8.36	0.872	1.65	87.4
GSR-only input	7.14±0.6598	9.92	0.821	1.91	84.6
Without fourier encoding	4.18±0.9359	6.21	0.928	1.28	91.5
Without Bi-GRU encoder	5.36±0.4512	7.84	0.889	1.52	89.2
Without attention pooling	5.82±0.3681	5.98	0.901	1.16	88.7
Without physiologically informed loss	4.47±1.2350	6.73	0.914	1.34	90.6
Proposed PI-Uncertainty fourier Bi-GRU-NN	3.09±0.9110	4.72	0.960	1.00	94.0

### 4.1 Discussion

Three findings stand out. First, fusing PPG and GSR with a Fourier-encoded attentive Bi-GRU produces strong held-out split performance in Table 4 and improves over the same-dataset baselines in Table 6, suggesting that multimodal sensing and frequency-aware encoding capture information that single-modality or raw-feature regressors may miss. Second, the composite regularized loss yields a smooth, well-behaved mapping—evidenced by the rapid, stable convergence in Figure 4, the tight error distribution in Figure 6, and the near-zero Bland–Altman bias in Figure 8—without imposing any artificial ordering on the data. Third, and most consequential for deployment, the subject-independent evaluation in Section 3.5 shows that inter-subject variability, rather than sample-wise noise, is the dominant remaining source of error: the MAE roughly doubles and $R^{2}$ drops by about 0.08 when moving from interpolation among known subjects to extrapolation to unseen subjects. The participants overlap can make a sample-wise split measure interpolation among already observed participants rather than generalization to new participants. The broad calibrated intervals show that the present model does not provide clinically precise estimates, and LOSO provides appropriate evidence of unseen-participant performance.

#### 4.1.1 Participant-independent model attribution of PPG and GSR

To examine attribution stability under subject-level distribution shift, the attribution analysis was repeated separately within each of the ten LOSO folds. For each held-out participant, absolute feature attributions were aggregated within the PPG and GSR modalities and normalized so that the two modality totals summed to 100%, as shown in as in Figure 15. Across the ten folds, PPG accounted for a mean ± sample SD of 90.01±2.15% of the normalized model attribution (range: 85.24%–92.07%), whereas GSR accounted for 9.99%±2.15% (range: 7.93%–14.76%). Thus, the fitted model was PPG-dominant for every held-out participant. This consistency describes the model’s reliance on its inputs; it must not be interpreted as a causal or biological partition of glucose-related information, nor does it by itself demonstrate an incremental benefit of GSR. Feature-attribution values describe how the fitted network distributes predictive influence among its inputs; they do not establish that either modality is responsible for a corresponding fraction of glucose-related information. We therefore interpret all percentages below exclusively as model attribution under the stated dataset, quality gate, and evaluation protocol. Although the GSR contribution is lower, it remains meaningful because GSR reflects electrodermal activity associated with sweat-gland responses and sympathetic nervous system modulation. Therefore, GSR provides complementary autonomic information that may help the model under specific physiological conditions such as stress, hydration variation, or altered skin conductance (8, 9, 21). The modality distribution, therefore, confirms that the proposed framework is PPG-dominant but not PPG-exclusive. Instead, it exploits PPG as the principal hemodynamic predictor and GSR as a supplementary electrodermal modality. This interpretation is consistent with the ablation study, where the fused PPG–GSR model outperformed the PPG-only and GSR-only variants, confirming that multimodal fusion improves the predictive accuracy of non-invasive BGL estimation (10, 12, 24).

**Figure 15:**
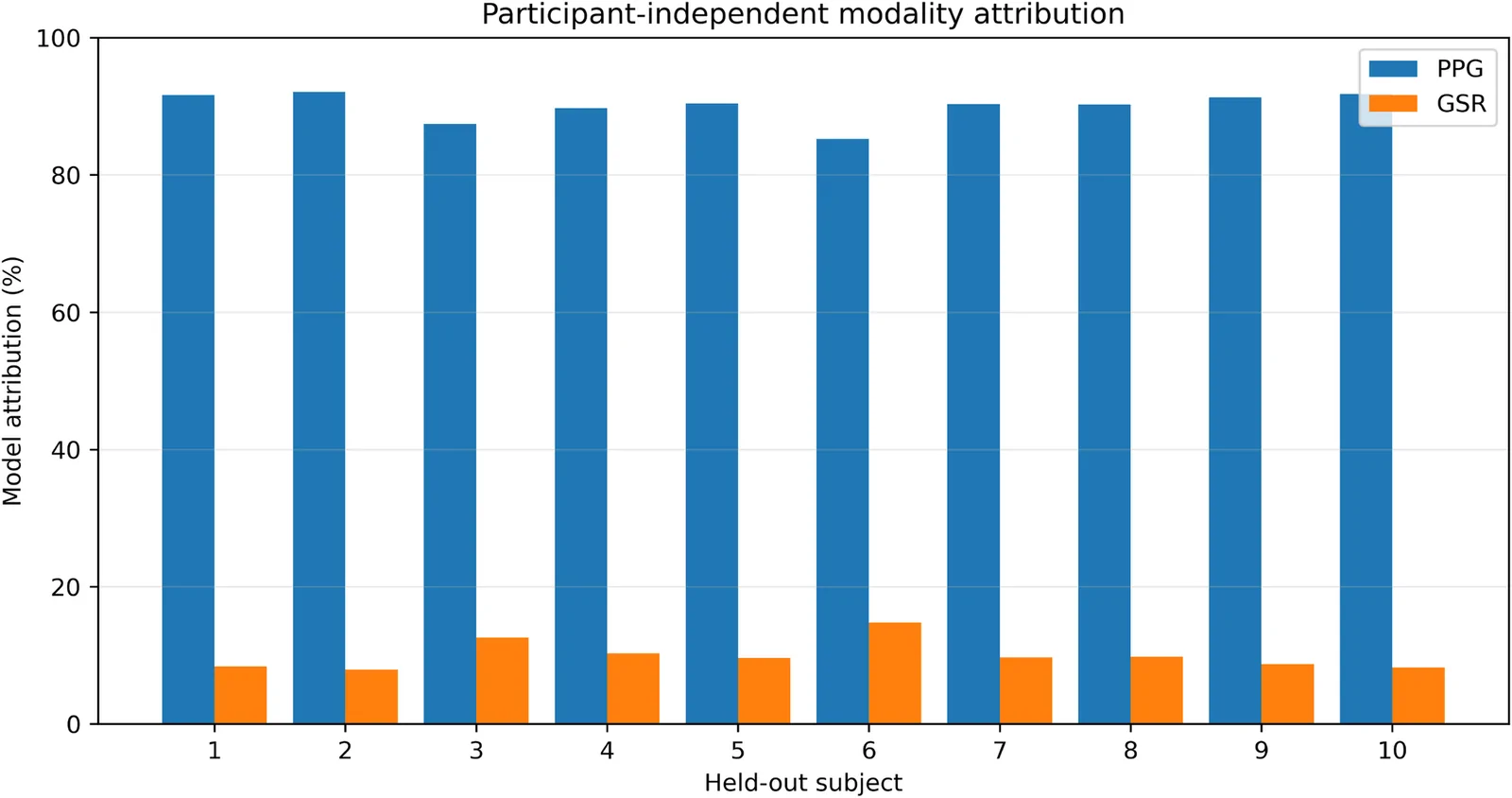
Participant-independent modality attribution across the ten LOSO folds assigned to PPG and GSR.

## 5 Conclusion

This study investigated an uncertainty-aware, Fourier-encoded attentive bidirectional gated recurrent unit model for glucose estimation from photoplethysmography and galvanic skin response, with continuous glucose monitor readings used as reference values. On the random sample-wise split, the model achieved an MAE of 3.09 mg/dL, an RMSE of 4.72 mg/dL, and $R^{2}=0.960$. In addition, 96.3% and 99.1% of the predictions fell in Clarke Error Grid Zone A and Zones A+B, respectively. These results and the ablation experiments suggest that multimodal fusion and Fourier feature encoding improve prediction within the evaluated cohort. However, because records from the same participants may occur in both sample-wise partitions, these results characterize within-cohort interpolation rather than generalization to new users. Under ten-fold leave-one-subject-out evaluation, the MAE increased to 16.751±0.435 mg/dL. This degradation demonstrates that intersubject variability and the limited representation of glycemic extremes remain the principal barriers to deployment.

Monte Carlo dropout provides the predictive center and an input-dependent dispersion estimate, while normalized split-conformal calibration constructs the final nominal 95% prediction intervals. The calibrated intervals achieved 95.66% empirical coverage with a mean width of 14.71 mg/dL on the sample-wise evaluation and 90.63% coverage with a mean width of 51.48 mg/dL across the pooled LOSO predictions. The LOSO undercoverage indicates that calibration based on the training participants does not fully transfer to an unseen participant under inter-subject distribution shift. Future work should therefore evaluate the method in larger and more diverse cohorts using participant- and session-disjoint protocols and should investigate calibration procedures designed specifically for cross-participant distribution shift.

## Data Availability

The raw data supporting the conclusions of this article are available from the corresponding author upon reasonable request.
